# Self-Assembled Peptide-Based Fibrous Hydrogel as a Biological Catalytic Scaffold for Nitric Oxide Generation and Encapsulation

**DOI:** 10.1021/acsami.5c03250

**Published:** 2025-04-29

**Authors:** Muhammad Younis, Tanveer A. Tabish, Cherly Firdharini, Mohamed Aslam, Mostafa Khair, Dalaver H. Anjum, Xuehai Yan, Manzar Abbas

**Affiliations:** † Department of Chemistry, Khalifa University of Science and Technology, 127788 Abu Dhabi, United Arab Emirates; ‡ Division of Cardiovascular Medicine, Radcliffe Department of Medicine, British Heart Foundation (BHF) Centre of Research Excellence, University of Oxford, Oxford OX3 7BN, U.K.; § Core Technology Platforms, New York University Abu Dhabi, 129188 Abu Dhabi,United Arab Emirates; ∥ Department of Physics, Khalifa University of Science and Technology, 127788 Abu Dhabi, United Arab Emirates; ⊥ University of Chinese Academy of Sciences, Beijing 100049, P. R. China; # State Key Laboratory of Biochemical Engineering, Institute of Process Engineering, Chinese Academy of Sciences, Beijing 100190, P. R. China; ¶ Functional Biomaterial Group, Khalifa University of Science and Technology, 127788 Abu Dhabi, United Arab Emirates

**Keywords:** peptides, self-assembly, noncovalent interactions, hydrogel, NO generation and encapsulation, anti-inflammatory

## Abstract

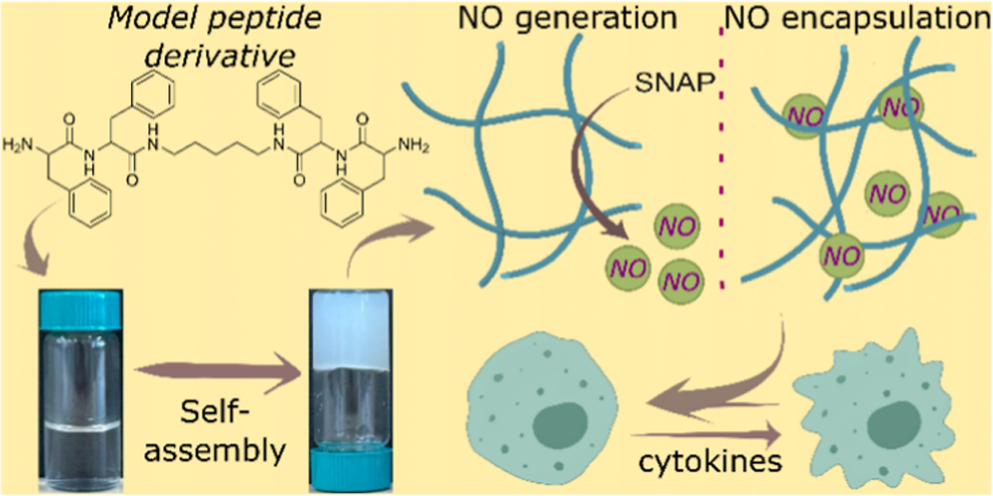

Biomolecular hydrogels are promising scaffolds for biomedical applications ranging from controlled drug release to personalized medicine. However, existing macromolecular scaffolds for nitric oxide (NO) release face several challenges, such as a low payload capacity, rapid release, and limited biocompatibility. Here, we present the design of short peptide derivatives as low-molecular-weight gelators that spontaneously self-assemble into nanofibrous hydrogels under basic aqueous conditions. Hydrogen bonding and hydrophobic interactions are central driving forces for the assembly process and contribute to tuning the mechanical properties. The nanofibrous hydrogel exhibits secondary structure properties, and the nanofibers show crystalline behavior. The terminal primary amines in the peptide building blocks could act as nucleophiles, facilitating the endogenous generation of NO gas, thus making the hydrogel scaffold a catalyst. The nanofibrous hydrogels can sequester NO from an external source that could be trapped in the interstices of the entangled fibrous networks. Simultaneously, it demonstrates anti-inflammatory effects in activated murine macrophages. This designer peptide hydrogel for NO generation and encapsulation provides fundamental insights into the design of peptide biomaterials for biomedical applications.

## 1 Introduction

Self-assembly is a bioinspired process that creates nano-to-microscale supramolecular materials and biomolecules, such as peptides and proteins, which spontaneously form nanofibrous structures through weak molecular interactions.^1−4^ Biomolecular hydrogels exhibit self-healing and thixotropic properties, which are required for various biomedical applications.^5^ Their tunable mechanical properties, external responsiveness, biocompatibility, and biodegradability make them attractive three-dimensional scaffolds.^6−9^ Hydrogels typically contain more than 90% water and structurally mimic the extracellular matrix of cells.^10^ Following the discovery of the first peptide hydrogel in 1993, several small-molecular-weight gelators containing amino acids and short peptides have been reported to construct nanofibrous hydrogels for applications in tissue regeneration, wound healing, and cardiac ischemia.^11−14^ However, individual amino acids and short peptides are generally unable to self-assemble to form hydrogels independently. Therefore, the covalent modification with non-natural amino acids, functional groups, and aromatic anchoring groups could tune their physicochemical properties to form ordered, smart nanofibrous hydrogels.^15−18^ Besides the covalent modification, factors such as concentration, pH, and solvents can also influence the gelation process and introduce the stimulus-responsive properties in peptide nanomaterials and hydrogels.^19−21^

NO is a therapeutic gaseous molecule that has been utilized to treat cardiovascular diseases for over 140 years, primarily in the form of organic nitrites and nitrates (glyceryl trinitrate), due to its vasodilatory, anti-inflammatory, antithrombotic, and angiogenic effects.^22−25^ However, these NO donors (organic nitrates) have limited therapeutic efficacy due to their short half-life, high NO diffusion rate, and scavenging in the presence of oxygen. These limitations make NO applications inadequate for the targeted, selective, and safe treatment of diseases in clinical practices. Various NO-delivering carriers and donor agents have been developed to overcome these challenges.^26^ Unfortunately, most of these donors and carriers suffer from low stability and limited NO payload capacity. Furthermore, although these catalysts produce NO in the blood circulation, they lack the specificity at the cellular level for targeted disease treatment.^27^ Therefore, to safely deliver NO at the required concentrations for therapeutic purposes, delivery vehicles are needed to precisely target the site of action with an accurate amount at the right time. It is important to know that the concentrations of the NO gas play a central role in the treatment. For example, 0.1 to 5 nM concentrations are used for physiological processes such as cell proliferation, neurotransmission,^28^ and vasodilation. The enzyme nitric oxide synthase (NOS) facilitates NO production through the oxidation of l-arginine. The efficacy of NO against diseases depends on the concentrations and release profile over time, that is, how long it is generated at the targeted site and at what concentration.^29^ Moreover, increased and controlled NO production at the micromolar level could yield fascinating results in the treatment of cancer and bacterial infections.^24^ However, the controlled release of NO and finding its biological catalytic agents are significant challenges for using this medical gas in biomedical applications.

Polymers such as chitosan and others have been widely studied as building blocks for hydrogel scaffolds, which serve as NO catalysts and delivery platforms (via encapsulation), due to their excellent hydrophilicity, biocompatibility, and three-dimensional (3D) nanostructures.^30−33^ Thermochemical or photochemical approaches have been employed for NO generation using donor *S*-nitroso glutathione (GSNO) encapsulated in pluronic F127 hydrogels, which act as catalysts in targeted cells.^34^ In another study, Nie and co-workers reported a NO-generating hydrogel matrix that promotes endothelial differentiation of mouse embryonic stem cells, providing a safe and valuable source for treating vascular diseases.^35^ Similarly, Lu and co-workers developed a hydrogel-based microneedle system with graphene oxide and GSNO, capable of NO release that could be triggered with light, making it applicable for wound healing.^36^ Generally, catalysts for NO generation and delivery vehicles have been developed using two-dimensional (2D) materials, such as graphene, and nanostructures like polymeric hydrogels and silica-based microspheres.^37^ Recently, peptide-based self-assembled nanomaterials, including metal coordination hybrid supramolecular nanomaterials, have been widely explored for anticancer and antibacterial applications.^38,39^ Therefore, dipeptide diphenylalanine (FF) was used in the formulation of the delivery platform with graphene oxide, and the main purpose of FF was to enhance biocompatibility and enable controlled delivery of NO.^40^ However, no hydrogel/material is formed from biologically relevant compounds such as peptides, which could explicitly serve as both (1) a catalyst to produce the NO from endogenous sources found in the bloodstream and (2) a carrier to sequester NO gas from an external source. The key challenges of biocompatibility, biodegradability, unreacted components in conjugation chemistry, and compromised toxicity that hinder nanomaterials from being considered for NO generation in healthcare applications must be addressed. The ideal solution is to design peptides for hydrogel formation and their utilization for NO generation and encapsulation.

**Figure 1:**
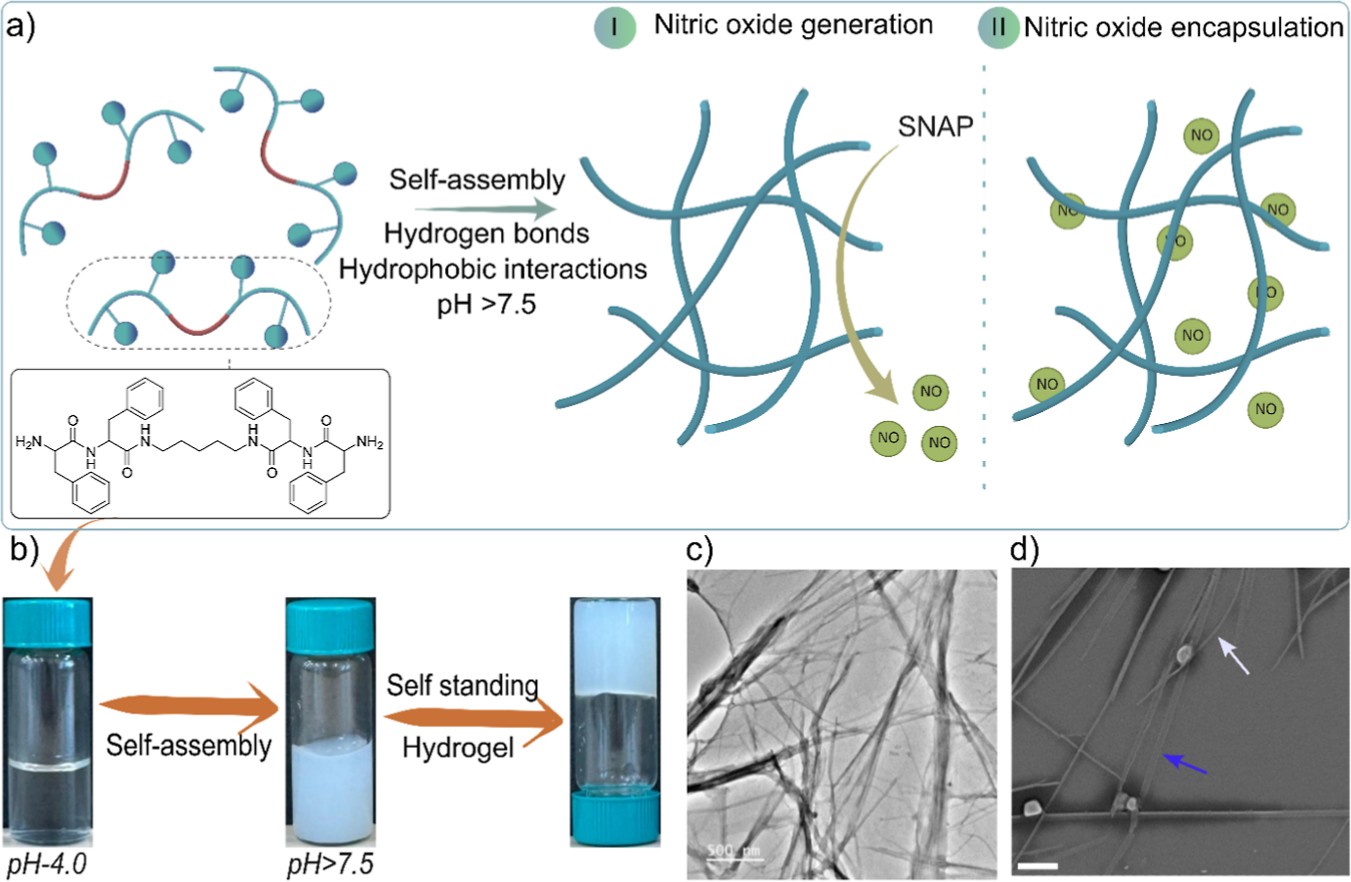
Schematic illustration for the self-assembled nanofibrous hydrogel for nitric oxide (NO) generation and encapsulation, (a) chemical structure of model compound (FFc_5_FF) and nitric oxide (NO) generation and encapsulation, (b) photographs of glass vials to show self-standing hydrogel formation at 10 mg/mL with Tris buffer (200 mM)–pH 8.0, (c) TEM image of the nanofibrous hydrogel at a concentration of 5 mg/mL of FFc_5_FF using Tris buffer (200 mM)–pH 8.0, and (d) SEM image of the nanofibrous hydrogel at the concentration of 1 mg/mL of FFc_5_FF using Tris buffer (200 mM), pH 8.0; the blue arrow indicates the bundle formation, while the white arrow indicates single nanofibers; the scale bar in (d) is 200 nm.

In this study, we rationally designed and synthesized the peptide derivatives through the sticker spacer approach, where the spacer (linker-c_5_) is hydrophobic and the hydrophobicity of the stickers varies with the constitutive amino acids. This rational design (Figure 1a) provides the structural features for the self-assembly of building blocks to form hydrogels through weak inter- and intramolecular interactions, mainly hydrogen bonding and hydrophobic interactions, as confirmed by molecular dynamics simulations. The fibrous hydrogel exhibits crystalline morphology, in contrast to the lyophilized powder of pristine peptide building blocks. To study the role of the hydrogel scaffold as a catalyst for generating NO, we exploited the *S*-nitroso-*N*-acetylpenicillamine (SNAP) donor reagent, and NO detection was performed by using spectroscopic and sensing techniques. In addition to its generation capability, the nanofibrous network in the hydrogel was able to sequester the NO gas from an external source. Furthermore, the macrophage cell line RAW 264.7 was used to test the anti-inflammatory properties of the NO-encapsulated nanofibrous hydrogel by measuring the release of cytokines, such as IL-1 and TNF-α.

## 2 Results and Discussion

### 2.1 Molecular Design of Sticker–Spacer Peptide Derivatives for Hydrogelation

We designed and synthesized the peptide derivatives using the sticker–spacer–sticker approach, incorporating aromatic amino acids (stickers) and hydrophobic linkers (spacers), through a solution-phase peptide synthesis method. Contrary to previously reported sticker spacer peptide derivatives, these compounds could form self-assembled nanofibrous hydrogels. The designer model compound (FFc_5_FF) consists of two aromatic stickers, diphenylalanine (FF), linked through a linear five-membered carbon chain (c_5_)—cadaverine. The compound has two amine groups at the terminal, potentially making it soluble in water. Interestingly, both the hydrophobic spacer and stickers are indispensable for the gelation of small-molecular-weight compounds into the hydrogel. These regions are responsible for enhancing hydrophobic interactions of designer compound FFc_5_FF in comparison to the spacer disulfide bond (FFssFF).^41^ The apolar disulfide linker is central for liquid–liquid phase separation (LLPS), which has been considered a pathway for self-assembly.^42^ Previously, when we incorporated the tyrosine amino acids in the stickers to make them hydrophilic, the resultant YFc_5_FY compound exhibited a rapid liquid-to-solid transition^43^ and formed star-shaped microstructures with small needle-like morphology, resembling the “sea urchin” shapes observed for FUS droplets.^44^ However, with a slight change in the sticker design, FFc_5_FF undergoes the assembly of well-ordered nanofibrous hydrogel rather than droplets (liquid), which show transition to solids (microstructures), and such liquid-to-solid transition is typically reported in many biomolecular assemblies.^4,45,46^

After characterizing the building blocks (Figure S1a–c), lyophilized powder of the designer peptide FFc_5_FF at different concentrations (1 to 10 mg/mL) was dissolved in 800 μL of Milli-Q water, followed by the addition of 200 μL of Tris buffer (1 M), pH 8.0. This change in pH can trigger the noncovalent interactions and spontaneously lead to self-standing nanofibrous hydrogel formation (Figures 1b, S2a, and S4a). The second designer compound, LFc_5_FL, which has relatively less hydrophobic stickers, also formed the hydrogel upon adding Tris buffer under the same conditions (Figures S3a and S4b). However, its gelation is slightly slower than that of FFc_5_FF, particularly at low concentrations. This can be attributed to reduced hydrophobicity in stickers because phenylalanine residues were replaced with leucine amino acids. It is noteworthy that when we further decreased the hydrophobicity by replacing both phenylalanine motifs with leucine in the third compound (LLc_5_LL), surprisingly, no hydrogel formation occurred, even at concentrations up to 100 mg/mL under the same conditions (Figure S4c). This suggests that hydrophobic interactions play a crucial role in the molecular assembly and gelation process for the formation of hydrogels using dipeptides with aromatic regions.^11^ Additionally, when we evaluated the gelation behavior of all three compounds in HEPES (1 M, pH 7.5) and phosphate buffer (1 M, pH 7.5), only FFc_5_FF was able to form the self-assembled hydrogels (Figures S2bc and S3bc).

To further explore the different behaviors of LLc5LL, we compared it with LLssLL, which undergoes LLPS to form coacervate droplets in sodium hydroxide (NaOH).^41^ Interestingly, LLc_5_LL also formed liquid droplets in sodium hydroxide at relatively high pH and concentration (Figure S5a), indicating the role of buffers in directing either ordered or disordered assembly of peptide derivatives. It is well known that Tris buffer, as an aqueous physiological buffer, facilitates the assembly process and plays a fundamental role in tuning the morphology of the resulting material. This is due to its ability to balance the ions or charges, ultimately affecting the noncovalent interactions involved in material formation.^47^ Furthermore, although both LFc_5_FL and FFc_5_FF (1 mg/mL) formed a hydrogel in NaOH, LFc_5_FL exhibited more random aggregation, whereas FFc_5_FF formed a well-ordered nanofibrous hydrogel (Figure S5bc). As the terminal amine groups play a pivotal role in the self-assembly of sticker–spacer peptide derivatives, they make the resulting nanofibrous hydrogel more responsive to pH. Interestingly, when we decreased the pH of the hydrogel by adding hydrochloric acid (HCl), we observed a transparent solution (Figure S6a). However, when the pH was increased above 12, random aggregates formed, appearing less self-organized and lacking the nanofiber structure (Figure S6bc). The hydrogel remains stable under physiological conditions, including typical pH and temperature (Figure S6d), which opens avenues for biomedical applications.

Since both the peptide derivatives FFc_5_FF and LFc_5_FL formed hydrogels in Tris buffer, we performed further morphological characterization at two different concentrations (1 and 5 mg/mL) using a transmission electron microscope (Figures 1c and 7a–e) and a scanning electron microscope for morphology investigations (Figure 1d). The nanofibers were more flexible at a 1 mg/mL concentration with a 20 ± 2 nm diameter. At a 5 mg/mL concentration, the size of nanofibers remained the same along with the bundling of nanofibers, where the size of the bundled fiber (blue arrow) is almost twice that of a single nanofiber (white arrow), as observed in the SEM image (Figure 1d). Interestingly, at higher concentrations and after aging the hydrogel for 24 h, we observed a shortening in the length of nanofibers and a slight morphology change into nanorods, particularly in LFc_5_FL. To confirm this change in morphology, we added urea to the hydrogel and observed no significant change in the nanofiber morphology (Figure S7f). The subtle change could be attributed to hydrogen bonding, which becomes stronger over time, and increasing the concentration of peptide derivative, resulting from decreasing the aromaticity (fewer phenylalanine residues) in the chemical design.^48,49^

### 2.2 Molecular Interactions Involved in the Self-Assembly of the Peptide Hydrogel

**Figure 2:**
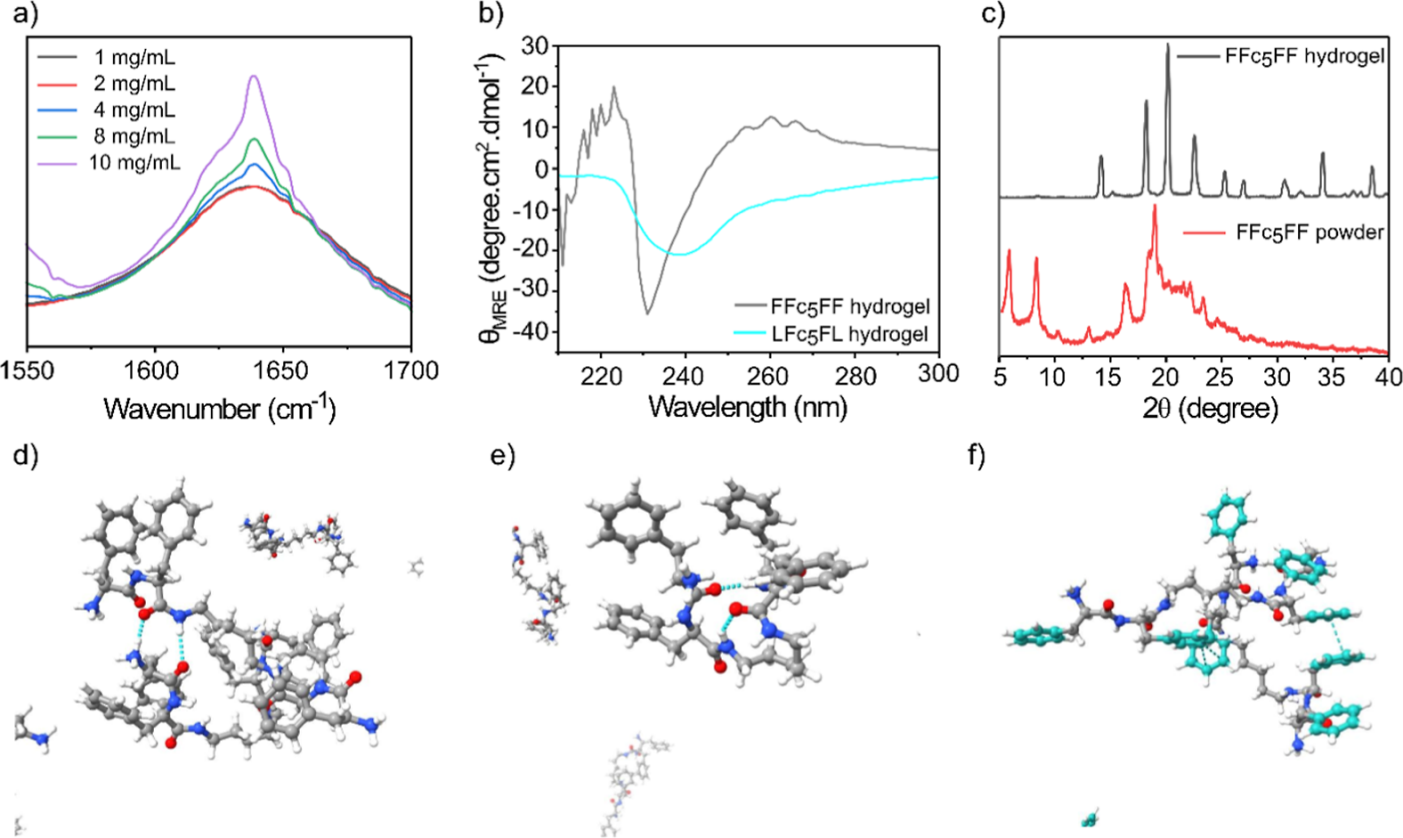
Spectroscopic characterization of the peptide hydrogel and molecular dynamics (MD) simulation, (a) FTIR spectra at different concentrations of FFc_5_FF, (b) circular dichroism spectra of FFc_5_FF and LFc_5_FL at 5 mg/mL after 2 h of preparation, (c) powder XRD patterns of FFc_5_FF at a 5 mg/mL concentration of the peptide lyophilized powder and hydrogel freeze-dried sample, (d) intermolecular hydrogen bonding shown by dotted bonds, (e) intramolecular hydrogen bonding shown by dotted bonds, and (f) hydrophobic interactions between aromatic regions.

We first conducted Fourier transform infrared (FTIR) spectroscopy analysis of the sticker–spacer-based hydrogel to gain more insight into molecular interactions involved in gelation. The most sensitive spectral region, amide I, provides the secondary structure information in proteins and peptides.^50^ This region corresponds to the stretching vibration of carbonyl (C=O) groups, typically ranging from 1600 to 1690 cm^–1^. FTIR spectroscopy analysis of freeze-dried hydrogels of FFc_5_FF and LFc_5_FL at different concentrations (1 to 8 mg/mL) revealed the C=O stretching of amide I at 1638 cm^–1^ and 1632 cm^–1^, respectively. At higher concentrations of peptide derivatives, the absorption peak becomes sharp (Figures 2a and S8a,b), suggesting the parallel arrangement of molecules in forming the β-turns secondary structures.^51^ The absence of a typical peak around 1690 cm^–1^ in the FTIR spectra further supports a disfavored antiparallel arrangement in molecular assembly, confirming the parallel arrangement of building blocks in the self-assembled hydrogel.^52^ The intensities of NH and OH stretching peaks are expected to increase when NH and OH groups participate in hydrogen bonding.^53^ Therefore, as the concentrations of FFc_5_FF and LFc_5_FL (Figure S8c) increased from 1 to 10 mg/mL, the NH stretching at 3287 cm^–1^ and 1692 cm^–1^ was enhanced, indicating hydrogen bonding in the hydrogel formation.^54^ The secondary structures of nanofibers in hydrogels formed by FFc_5_FF and LFc_5_FL were further investigated by circular dichroism (CD) spectroscopy. At 5 mg/mL, we observed the positive Cotton effect and then the characteristic peak at 230 for FFc_5_FF in the negative region, while at the same concentration, the negative Cotton effect was observed. The characteristic peak at 238 nm for LFc_5_FL in the negative region suggests the parallel arrangement of building blocks to form β-turn conformations in nanofibers^55^ (Figure 2b), consistent with the FTIR results.

To further delve into the molecular interactions and structural properties, we performed powder X-ray diffraction (XRD) and observed two distinct peaks at 5.80° and 8.23° in FFc_5_FF lyophilized powder, indicating an amorphous nature. However, these peaks were absent in the XRD pattern of the peptide hydrogel, and three prominent peaks appeared that corresponded to 18.32°, 20.27°, and 25.37°, indicating structural transformation into a crystalline form upon gelation of the peptide derivative (Figure 2c). Furthermore, we investigated the molecular interactions responsible for hydrogel formation using molecular dynamics simulations. The results revealed that both the inter- and intramolecular hydrogen bonding are essential for hydrogel formation. Specifically, two intermolecular hydrogen bonds with lengths of 1.8 Å and 2.22 Å, as well as two intramolecular hydrogen bonds of 1.87 Å and 2.16 Å, were identified, labeled as cyan color dotted bonds in Figure 2d–e. In addition to hydrogen bonding, hydrophobic interactions, mainly from the aromatic regions of FFc_5_FF, also contribute to hydrogel formation (Figures 2f and S9ab). Interestingly, the FFc_5_FF compound also formed hydrogen bonds with the water molecules in the hydrogel (Figure S10ab). A small cluster of three molecules formed by intermolecular hydrogen bonds exhibited bond energies of −7.01 and −8.58 kcal/mol at 36 ns, while hydrogen bonds between water molecules and the peptide motif exhibited average bond energies of −8.29 kcal/mol (Figure S11).

### 2.3 Mechanical Properties of the Peptide Hydrogel

**Figure 3:**
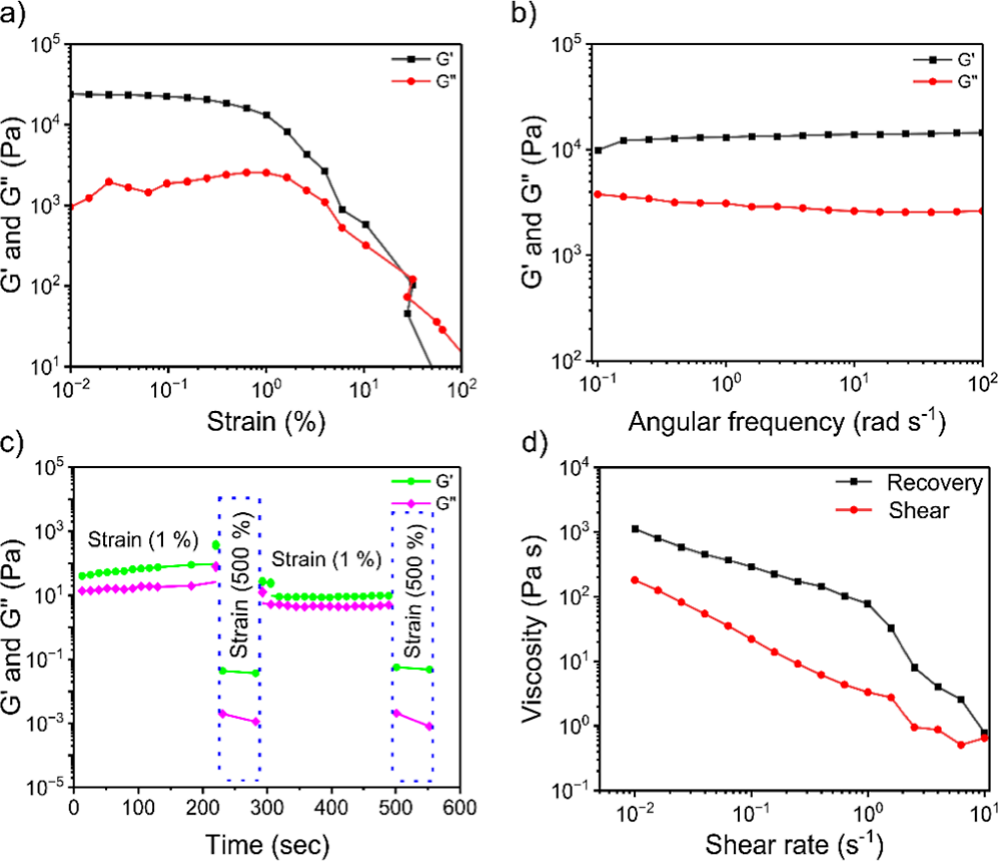
Rheological characterization of the peptide-based hydrogel, (a) strain-dependent (ω = 10 rad s^–1^) oscillatory shear rheology of the FFc_5_FF hydrogel (10 mg/mL), (b) angular frequency-dependent oscillatory shear rheology of the FFc_5_FF hydrogel (10 mg/mL) at a strain of 1%, (c) the evaluation of self-healing property of the FFc_5_FF hydrogel using the continuous step-strain measurements, at 1% and 500% oscillatory strain for two cycles, and (d) shear thinning and rapid recovery, as observed from the viscosity of the FFc_5_FF hydrogel at increased shear rate during a continuous flow experiment.

Thixotropic and mechanical properties are crucial for the injectability of hydrogels, which are essential for drug encapsulation and sustained release for biomedical applications.^56,57^ Therefore, we evaluated the mechanical properties, such as shear thinning and self-healing, of hydrogels formed at 10 mg/mL concentrations of FFc_5_FF and LFc_5_FL (Figure S12a–c) in Tris buffer. These properties were assessed by investigating the elastic or storage modulus (*G*′) and loss or viscous modulus (*G*″). The strain-dependent oscillatory rheology of the FFc_5_FF hydrogel demonstrates viscoelastic properties with exceptional antishear ability, indicating shear thinning behavior, and the hydrogel network remains intact at high strain until 30% (Figure 3a). In addition, frequency-dependent oscillatory shear rheology of the FFc_5_FF hydrogel at a constant strain of 1% revealed that *G*′ is higher than *G*″ across the entire observed frequency range, indicating typical viscous behavior of the hydrogel (Figure 3b). To further evaluate the self-healing properties of the hydrogel, we performed the continuous step change of the oscillatory strain between 1 and 500% at a frequency of 1 rad/s. This test assesses the strain-induced damage and self-healing properties of the FFc_5_FF hydrogels (Figure 3c). At higher strains (500%), *G*′ and *G*″ decreased to minimum values, suggesting the breakage of the hydrogel network. However, when a low strain (1%) was applied, both *G*′ and *G*″ recovered, demonstrating the restoration of the hydrogel network. These results show that the broken structure rapidly recovers after being broken and displays normal hydrogel behavior after two cycles of applying high and low strains, confirming its excellent shear-thinning behavior. The recovery profile of the FFc_5_FF hydrogel was also measured by gradually decreasing the strain from 10% to 0.01%, demonstrating that the viscosity of the hydrogel returns to its initial state (Figure 3d). Based on these results, we conclude that the hydrogel is primarily formed and stabilized by weak noncovalent interactions, which likely contribute more to its higher viscosity compared with covalently cross-linked polymer hydrogels. This reversible nature imparts tunable mechanical properties, making it a promising scaffold for biomedical applications due to its self-healing and excellent shear-thinning properties.

### 2.4 Nitric Oxide Generation, Encapsulation, and Anti-inflammatory Properties

**Figure 4:**
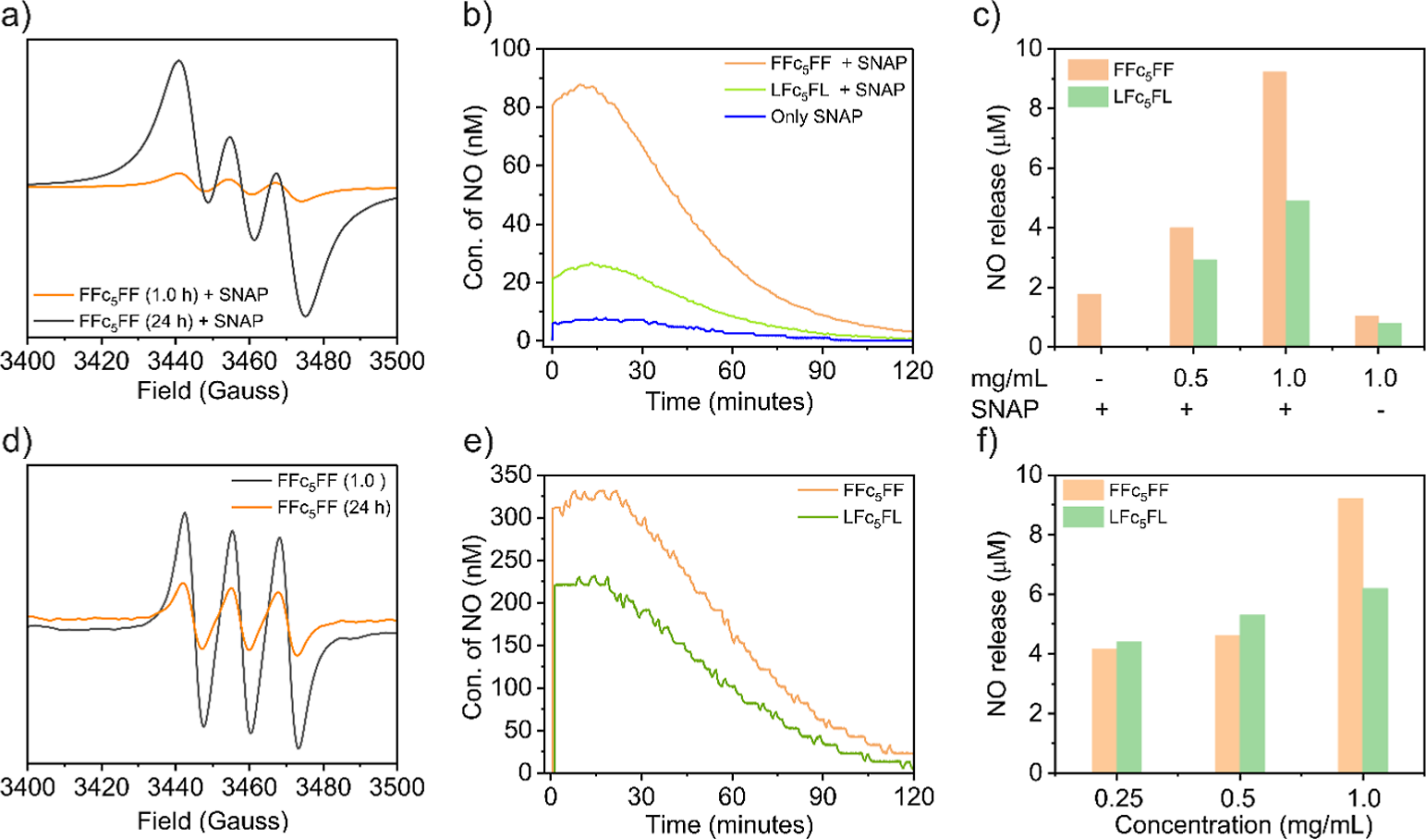
NO generation and encapsulation characterization, (a) EPR spectra for NO generation from the FFc_5_FF nanofibrous hydrogel as the catalyst (1 mg/mL, 1.0 and 24 h aged) with SNAP (10 mM), (b) electrochemical sensor detection of NO generation from the FFc_5_FF and LFc_5_FL hydrogels (1 mg/mL) with SNAP (10 mM), (c) chemiluminescence detection of NO generated from the FFc_5_FF and LFc_5_FL hydrogels at a concentration of 0.5 and 1 mg/mL, (d) EPR spectra of NO encapsulated in the FFc_5_FF nanofibrous hydrogel (1 mg/mL, 1.0 and 24 h aged), (e) electrochemical sensor detection of NO encapsulation in the FFc_5_FF and LFc_5_FL hydrogel at 1 mg/mL, (f) chemiluminescence detection of the NO encapsulation in FFc_5_FF and LFc_5_FL hydrogel scaffolds at concentrations of 0.25, 0.5, and 1 mg/mL. All samples were prepared in Tris buffer (pH 8.0, 200 mM). The data for bar graphs (c,d) of chemiluminescence is extracted from the graphs in Figure S16a,b.

NO is a free radical gas, a key signaling molecule produced transiently by the nitric oxide synthase (NOS) enzyme in mammalian cells. It plays a variety of physiological roles in living systems, including vasodilation, platelet aggregation, antibacterial activity, and the regulation of inflammation.^58^ In relation to the hypothesis of this research, we first conducted electron paramagnetic resonance (EPR) experiments, a more accurate spectroscopic method for detecting NO generation from the nanofibrous hydrogel, either indigenously or encapsulated and then released from the hydrogel matrix. The NO generation could be detected through the paramagnetic properties of NO due to the Zeeman effect arising from the nitrogen atoms of amine groups in building blocks. For detection, we used a (DETC)_2_Fe complex to trap NO generated from FFc_5_FF and LFc_5_FL nanofibrous hydrogels (1 mg/mL, pH 8.0, 200 mM Tris buffer), producing the EPR active spin adduct NO-(DETC)_2_Fe. The characteristic three-line isotropic EPR spectrum of NO-(DETC)_2_Fe demonstrates that NO gas is generated from the 1 h aged nanofibrous hydrogel of both FFc_5_FF and LFc_5_FL compounds in the presence of SNAP (10 mM). Interestingly, the signal was detected after 24 h with a lower intensity (Figures 4a and S13a); this decrease in the intensity could be attributed to the involvement of amine groups in the hydrogen bonding, thus making them less available as a nucleophile to react with SNAP (the electrophile). To exclude any signals from the spin-trap adduct or SNAP, we performed the EPR of both separately and found no detection of NO gas (Figure S14ab). It is worth noting that the Tris buffer used for hydrogel formation and monomers of peptide derivative (FFc_5_FF), without gelation, do not generate NO gas. No EPR signal was detected in the spectra when we used buffer and monomers alone under the same conditions (Figure S15ab). This demonstrates that the primary amines of peptide derivatives in the protonated form act as nucleophiles to react with SNAP (electrophile) and produce the NO gas because amine (NH_2_) protonation occurs under basic pH conditions.

Taking it a step further, we employed an electrochemical sensor to monitor the real-time generation of NO gas, which was approximately 80 and 20 nM from 1 mg/mL nanofibrous hydrogel of FFc_5_FF and LFc_5_FL, respectively, in the presence of SNAP (Figure 4b). These findings confirm the catalytic role of the hydrogels. The release profiles show that NO gas can be generated from the nanofibrous hydrogel within 1 h, with SNAP being used as an electrophile (donor). To validate the NO generation confirmed by both EPR results and the NO analyzer, we used ozone-based chemiluminescence to measure NO generation from FFc_5_FF and LFc_5_FL nanofibrous hydrogels with two concentrations (0.5 and 1 mg/mL), which generate continuous NO gas upon adding the SNAP (10 μM) (Figure 4c). The NO generation (approximately 9.20 μM) from the FFc_5_FF nanofibrous hydrogel was considerably higher than the (approximately 6.30 μM) LFc_5_FL nanofibrous hydrogel, even though both hydrogels were used at the same concentrations (1 mg/mL). This subtle difference in NO generation between the two different hydrogels could be due to the difference in the availability of amine groups as nucleophiles. Notably, neither the nucleophiles (hydrogels) nor the electrophiles (SNAP) alone produce a considerable quantity of NO gas, suggesting that a catalytic reaction is indispensable for NO generation. Based on the results obtained from EPR, electrochemical sensors, and chemiluminescence techniques, we emphasize that the sticker–spacer peptide-based hydrogel scaffold could serve as an excellent catalyst for the endogenous generation of NO gas.

After successfully confirming the catalytic ability of the peptide hydrogel to generate NO, it was essential to investigate whether these three-dimensional hydrogel networks could also encapsulate NO gas from an external source and release it on demand or store a high concentration of NO if needed for biomedical applications. We utilized characterization techniques such as EPR, electrochemical sensors, and chemiluminescence to investigate the encapsulation capability of hydrogels (Figure 4b). To this end, we encapsulated NO gas in the nanofibrous hydrogels of FFc_5_FF and LFc_5_FL (1 mg/mL), and the EPR results confirmed that the gas is sequestered within the interstices of nanofiber scaffolds (Figures 4d and S13b). Interestingly, after 24 h of encapsulation, the characteristic signals of NO gas were still detectable, but the signal intensity had significantly decreased, which could be attributed to air leakage. Moreover, when we harnessed an electrochemical sensor for the delivery profiles of NO gas, it was observed that FFc_5_FF and LFc_5_FL hydrogels can encapsulate approximately 300 nM and 210 nM gas concentrations, respectively (Figure 4e). This demonstrates that more hydrophobic interactions in FFc_5_FF make it a more attractive delivery platform compared to its analogue peptide derivative, LFc_5_FL. Besides this, at three different concentrations (0.25, 0.5, and 1 mg/mL) of FFc_5_FF and LFc_5_FL, we noticed that NO gas is successfully encapsulated inside the nanofibrous network, as confirmed by chemiluminescence. FFc_5_FF nanofibers exhibited a higher capability compared to LFc_5_FL nanofibers (Figure 4f). Our designer sticker–spacer peptide derivatives, which form hydrogels, have shown the ability to sequester the NO gas and could serve as the delivery vehicle for delivering medical gases (such as NO) to targeted sites.

**Figure 5:**
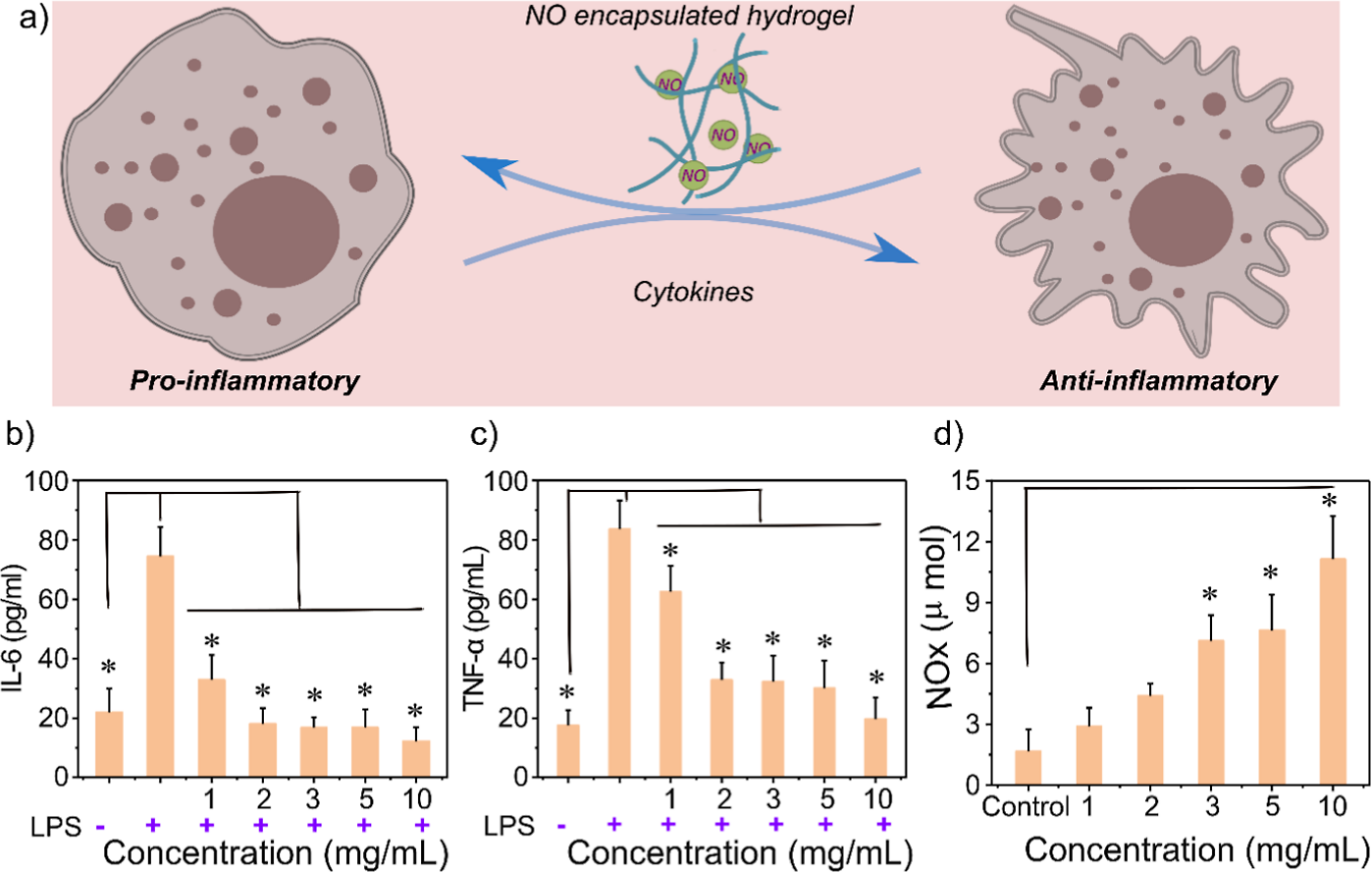
Anti-inflammatory studies, (a) schematic illustration, (b) proinflammatory cytokines IL-6, (c) TNF-α, enzyme-linked immunosorbent assay (ELISA), (d) Griess assay to measure the amounts of nitrites released from the FFc_5_FF nanofibrous hydrogel (different concentrations) in LPS-induced RAW264.7. Data is expressed as mean ± SD (*n*-3). *p < 0.05, vs (−) LPS in (b,c), while for the control group in (d), *p* values were calculated by analysis of variance (one-way ANOVA) with Tukey’s test.

NO is a small signaling molecule with a potent role in inflammatory and immune responses, regulating cytotoxicity against microorganisms.^59^ It reduces inflammatory cell recruitment, infiltration, and polarization and releases inflammatory cytokines during vessel wall injury.^60,61^ After investigating the NO-generating ability of peptide hydrogels, we evaluated whether this scaffold could release the NO (stored from an external source) in cells cultured under in vitro conditions, which provides insights into the mechanisms of NO release from the hydrogel scaffolds of both compounds and whether they show any effects on pro-inflammatory markers (Figure 5a). We selected RAW 264.7 macrophages, a well-characterized murine macrophage cell line, to demonstrate the anti-inflammatory actions of hydrogel scaffolds. These macrophages produce high levels of pro-inflammatory cytokines upon stimulation with lipopolysaccharides (LPS), an endotoxin that activates the inflammatory M1 pathway (Figure 5a). Two pro-inflammatory cytokine markers, IL-6 and TNF-α (Figures 5bc and S17ab), were measured with (M1 pathway activation) and without (M0 resting state) LPS stimulation across different hydrogel concentrations. The hydrogel significantly reduced the levels of both pro-inflammatory cytokines at higher concentrations. For instance, at 10 mg/mL, the expression of IL-6 was decreased to 17 pg/mL compared with 78% of untreated IL-6. Similarly, TNF-α levels were significantly reduced at higher doses of hydrogel scaffolds (Figure 5c). These findings indicate a dose-dependent anti-inflammatory effect of the hydrogel scaffolds. The NO_*x*_ (nitrite, a byproduct of NO) was quantified using the Griess assay (Figures 5d and S17c) to confirm that this effect arises from NO released from hydrogels. At a hydrogel concentration of 10 mg/mL, the nitrite produced was measured at 12 μmol, directly correlating with the observed inhibition of pro-inflammatory cytokines. This relationship suggests that NO released from the hydrogel scaffold modulates the inflammatory response by suppressing the release of IL-6 and TNF-α cytokines. These results demonstrate that the NO-generating nanofibrous hydrogel scaffold efficiently releases NO and exhibits remarkable anti-inflammatory effects by suppressing the levels of key pro-inflammatory cytokines in activated macrophages. This work provides insights into how NO released from hydrogels can mitigate inflammation, making them a promising platform for therapeutic applications targeting inflammatory conditions in wide-ranging human diseases.

## 3 Conclusions

In summary, we have developed a sticker–spacer peptide-based supramolecular hydrogel as a biological scaffold catalyst for NO generation, capable of sequestering the NO within the network of nanofibers in the hydrogel. Weak molecular interactions, such as hydrogen bonding and hydrophobic interactions, are involved in the self-assembly process of peptide derivatives in the nanofibrous hydrogel. Since the designer peptide derivative FFc_5_FF contains primary amine groups at the terminal, these act as nucleophiles that react with electrophiles, such as SNAP, to generate NO. The low payload capacity, fast release, and compromised biocompatibility are real challenges in the existing limited macromolecular scaffolds for nitric oxide generation and its release. Our designer nanofibrous hydrogels could overcome the aforementioned challenges. Furthermore, macrophages RAW 264.7 were used for pro-inflammatory cytokines (such as IL-1 and TNF-α) to test the anti-inflammatory properties of the NO-encapsulating hydrogel. Our designer hydrogels demonstrate considerable potential as biological scaffolds for medical gases such as NO encapsulation and targeted delivery, offering a promising approach for combating resistant bacterial strains and other applications.

## 4 Materials and Methods

### 4.1 Materials

Boc-l-phenylalanine (Boc-Phe-OH), Boc-l-leucine (Boc-Leu-OH), Boc-l-tyrosine (Boc-Tyr-OH), Boc-l-tryptophan (Boc-l-Trp-OH), 1-hydroxybenzotriazole (HOBt), 1 M Tris buffer, hexafluorophosphate azabenzotriazole tetramethyl uronium (HATU), and *N*,*N*-diisopropylethylamine (DIPEA) were purchased from Sigma-Aldrich. Cadaverine and 4 M hydrogen chloride solution in dioxane were obtained from Fluorochem. All solvents and salts were used as received, unless otherwise stated. The peptide derivatives were synthesized using solution-phase peptide synthesis,^41^ and detailed synthesis steps and characterization of building blocks are given in the Supporting Information.

### 4.2 Methods

#### 4.2.1 Preparation of Hydrogels

The lyophilized powder of LLc_5_LL, LFc_5_FL, and FFc_5_FF with different concentrations (1 to 10 mg/mL) was dissolved in 800 μL of water, and then 200 μL of Tris buffer (1 M) with pH 8.0 was added, which turned the peptide solutions into a nanofibrous hydrogel immediately, except for the LLc_5_LL compound. The aqueous solutions of FFc_5_FF (1–10 mg/mL) also produced a hydrogel when 200 μL of phosphate buffer (1 M) with pH 7.5 and HEPES buffer (1 M) with pH 7.5 were added instead of the Tris buffer. Similarly, a solution of LFc_5_FL with different concentrations (as FFc_5_FF) formed the hydrogel only in the Tris buffer.

#### 4.2.2 Microscopic Studies

##### 4.2.2.1 Optical Microscopy

An Olympus microscope with 40× and 100× objectives was used to acquire the images of LFc_5_FF and FFc_5_FF nanofibrous hydrogels at different concentrations ranging from 1 to 10 mg/mL (final concentration). The microscope was coupled with a high-resolution CAM-ORCA-FLASH4.0 V3 Digital Hamamatsu sCMOS camera.

##### 4.2.2.2 Scanning Electron Microscopy

A ten μL amount of FFc_5_FF hydrogel samples (1 and 5.0 mg/mL) was drop-cast on a clean silicon wafer using a micropipette, and the extra liquid was blotted with filter paper. All samples were dried overnight in a fume hood, and images were taken using the FEI Nova NanoSEM 650 at 10 kV. Before acquiring images, the samples were sputtered with Pt (nanosized film) by JEOL JEC-300FC Auto Fine Coater. The average diameter of nanofibers was calculated by processing the acquired SEM images with ImageJ and OriginPro 2023b software packages.

##### 4.2.2.3 Transmission Electron Microscopy Images

The holey carbon-coated copper grid (mesh size 300 nm) (purchased from Electron Microscopy Sciences) was immersed in freshly prepared FFc_5_FF (1 and 5.0 mg/mL) hydrogel samples for 30 s. Afterward, the sample containing grids was gently rubbed on the drops (50 μL) of the negative staining reagent, phosphotungstic acid. The TEM grid was then dried at room temperature before imaging the morphology of the nanofibrous hydrogel using ThermoFisher Scientific’s Talos 200 transmission electron microscope, equipped with a field emission gun. The experiments were performed by operating the microscope at a 200 kV accelerating voltage. Several bright-field TEM (BF-TEM) images were obtained by setting the microscope at different magnifications to understand the nanofiber’s morphology.

#### 4.2.3 Nitric Oxide Characterization

##### 4.2.3.1 Nitric Oxide Gas Encapsulation

To prevent the rapid reaction of NO with oxygen, samples were first purged with nitrogen (N_2_) gas to remove any dissolved oxygen. They were then placed and stored in 7 mL glass vials with a PTFE liner (27151 and 27157, Sigma-Aldrich, USA). Each sample was purged with a nitrogen headspace (BOC) for 5 min, followed by exposure to NO gas (400 ppm of NO in nitrogen, BOC, UK) for 5, 10, and 60 min. A needle was positioned just above the liquid surface to regulate gas flow and ensure uniform sample mixing before the measurement of NO release.

##### 4.2.3.2 Electron Paramagnetic Resonance

Electron paramagnetic resonance (EPR) was employed to detect NO release from the samples of FFc_5_FF and LFc_5_FL nanofibrous hydrogels (0.25, 0.5, and 1 mg/mL) in Tris buffer with pH 7.5. DETC_2_Fe was used as the NO trapping agent, as previously reported.^40^ To prepare the spin trap solution, NaDETC (250 mM, 5 mL) and iron(II) sulfate (FeSO_4_·7H_2_O, 50 mM, 5 mL) were dissolved separately in degassed Milli-Q water. The two solutions were combined in 10 mL of dichloromethane (CH_2_Cl_2_) to form a pale-yellow–brown opalescent DETC_2_Fe colloid. The spin trap solution was used immediately. Samples at a concentration of 250 μg/mL were incubated with the spin trap solution for 30 min. Subsequently, the solution’s aliquot (20 μL) was transferred into a quartz EPR tube and analyzed by X-band EPR spectroscopy. The three-line peak was obtained at room temperature by EPR, confirming the formation of the NO–DETC_2_Fe complex. EPR spectra were recorded with a conventional continuous wave (CW) homodyne microwave bridge and TE_011_ resonator by using a Bruker BioSpin EMXmicro spectrometer equipped with a premium X-band (9.1–9.9 GHz) source. The microwave frequency was set at 9.877 GHz, with a power output of 10 mW and a field modulation of 0.3 mT. Measurements were conducted at room temperature using 1.2 mm (internal diameter) clear fused quartz capillaries and nitrogen gas purge. Instrumental parameters normalized signal amplitudes, with samples filling the vertical height of the resonator. At 50 mM Fe(II)SO_4_ and 250 mM DETC, the nitrosyl iron signal was detected on the slope of a large ferromagnetic background signal from iron oxides. In the case of NO-generating samples, the concentrations of 250 μg/mL and SNAP (10 μM) were incubated with the spin trap solution for 30 min. Subsequently, the sample with an aliquot (20 μL) was transferred to a quartz EPR tube and analyzed by X-band EPR spectroscopy.

##### 4.2.3.3 Real-Time NO Measurement Using an Electrochemical Sensor

The real-time NO release was measured by using a free radical analyzer (TBR4100, World Precision Instruments) equipped with a NO-sensitive electrode (ISO–NOP, World Precision Instruments). Before taking the measurements, the NO electrode was polarized and calibrated according to the manufacturer’s instructions. Specifically, the electrode probe was submerged in a glass vial containing 10 mL of 0.1 M H_2_SO_4_ and 0.1 M potassium iodide to establish a baseline current measurement. A successive 25 μM NaNO_2_ solution was added to generate a calibration curve, creating a range of NO concentrations. The NO concentrations were calculated based on the stoichiometric conversion of sodium nitrite to NO. To detect the concentration of NO release from the samples of FFc_5_FF and LFc_5_FL nanofibrous hydrogels (0.25, 0.5, and 1 mg/mL), the NO probe was immersed in a glass vial filled with 4 mL of the peptide hydrogels in a Tris buffer at pH 7.5. The calibration curve was then used to determine the NO release based on changes in current responses. The glass vials were covered with aluminum foil and kept on a hot plate at 37 °C, with continual stirring throughout the NO measurement. In the case of evaluating the NO-generating capacity of peptide hydrogels, the samples at a concentration of (total volume of 100 μL) were added to the vial, and SNAP (10 μM) was added to the vial.

##### 4.2.3.4 Ozone-Based Chemiluminescence

The concentration of NO released from the samples of FFc_5_FF and LFc_5_FL nanofibrous hydrogel (0.25, 0.5, and 1 mg/mL) was added to the helium gas test solution and detected with a chemiluminescence NO analyzer (NOA) (Sievers 280, Boulder, CO). PBS −1 M (pH 7.4), comprising 0.5 mM EDTA (1:0.5), was prepared for the SNAP solution. A 10 μL (10 μM) aliquot of each sample was added into the vessel (with a constant helium gas flow) using a gastight syringe through the vessel’s injection unit. The purging vessel was covered with aluminum foil to prevent light exposure. Each measurement was continued until the NO spectrum reached the baseline levels. The calibration curve was obtained via sodium nitrite reduction in an acidified vanadium chloride solution. The amount of NO generated from the sample was determined from the calibration curves. Microsoft Excel 2010 (Microsoft, Redmond, WA, USA) and OriginPro 2023a were used to process raw data. In NO-generation measurements, a 10 μL aliquot of each sample was added into the purging vessel with and without SNAP (10 μM). Statistical analysis (one-way ANOVA) was performed using OriginPro 2023a.

## Supporting Information Available

Synthesis and characterization (^1^HNMR,^13^CNMR, and LC–MS) of peptide derivatives; photographs of hydrogels in Eppendorf and microscopic images; TEM images at different concentrations of FFc_5_FF and LFc_5_FL hydrogels; FTIR and CD experimental details, mechanical properties, XRD, and computational details; and EPR, electrochemical sensor, and anti-inflammatory results (PDF)

## References

[ref1] Sinha N. J.; Langenstein M. G.; Pochan D. J.; Kloxin C. J.; Saven J. G. Peptide Design and Self-Assembly into Targeted Nanostructure and Functional Materials. Chem. Rev. 2021, 121 (22), 13915–13935. 10.1021/acs.chemrev.1c00712. 34709798

[ref2] Hauser C. A.; Zhang S. Designer Self-Assembling Peptide Nanofiber Biological Materials. Chem. Soc. Rev. 2010, 39 (8), 2780–2790. 10.1039/b921448h. 20520907

[ref3] Zhao X.; Pan F.; Xu H.; Yaseen M.; Shan H.; Hauser C. A.; Zhang S.; Lu J. R. Molecular Self-Assembly and Applications of Designer Peptide Amphiphiles. Chem. Soc. Rev. 2010, 39 (9), 3480–3498. 10.1039/b915923c. 20498896

[ref4] Chang R.; Yuan C.; Zhou P.; Xing R.; Yan X. Peptide Self-Assembly: From Ordered to Disordered. Acc. Chem. Res. 2024, 57 (3), 289–301. 10.1021/acs.accounts.3c00592. 38232052

[ref5] Diba M.; Spaans S.; Ning K.; Ippel B. D.; Yang F.; Loomans B.; Dankers P. Y.; Leeuwenburgh S. C. Self-Healing Biomaterials: From Molecular Concepts to Clinical Applications. Adv. Mater. Interfaces 2018, 5 (17), 1800118 10.1002/admi.201800118.

[ref6] Kuang Y.; Shi J.; Li J.; Yuan D.; Alberti K. A.; Xu Q.; Xu B. Pericellular Hydrogel/Nanonets Inhibit Cancer Cells. Angew. Chem., Int. Ed. 2014, 53 (31), 8104–8107. 10.1002/anie.201402216. PMC4116430 24820524

[ref7] Fleming S.; Ulijn R. V. Design of Nanostructures Based on Aromatic Peptide Amphiphiles. Chem. Soc. Rev. 2014, 43 (23), 8150–8177. 10.1039/C4CS00247D. 25199102

[ref8] Sun T. L.; Kurokawa T.; Kuroda S.; Ihsan A. B.; Akasaki T.; Sato K.; Haque M. A.; Nakajima T.; Gong J. P. Physical Hydrogels Composed of Polyampholytes Demonstrate High Toughness and Viscoelasticity. Nat. Mater. 2013, 12 (10), 932–937. 10.1038/nmat3713. 23892784

[ref9] Chang R.; Zhao L.; Xing R.; Li J.; Yan X. Functional Chromopeptide Nanoarchitectonics: Molecular Design, Self-Assembly and Biological Applications. Chem. Soc. Rev. 2023, 52 (8), 2688–2712. 10.1039/D2CS00675H. 36987746

[ref10] Vernerey F. J.; Lalitha Sridhar S.; Muralidharan A.; Bryant S. J. Mechanics of 3d Cell–Hydrogel Interactions: Experiments, Models, and Mechanisms. Chem. Rev 2021, 121 (18), 11085–11148. 10.1021/acs.chemrev.1c00046. 34473466

[ref11] Smith A. M.; Williams R. J.; Tang C.; Coppo P.; Collins R. F.; Turner M. L.; Saiani A.; Ulijn R. V. Fmoc-Diphenylalanine Self Assembles to a Hydrogel Via a Novel Architecture Based on Π–Π Interlocked Β-Sheets. Adv. Mater. 2008, 20 (1), 37–41. 10.1002/adma.200701221.

[ref12] Gelain F.; Luo Z.; Zhang S. Self-Assembling Peptide Eak16 and Rada16 Nanofiber Scaffold Hydrogel. Chem. Rev. 2020, 120 (24), 13434–13460. 10.1021/acs.chemrev.0c00690. 33216525

[ref13] Lan T.; Dong Y.; Shi J.; Wang X.; Xu Z.; Zhang Y.; Jiang L.; Zhou W.; Sui X. Advancing Self-Healing Soy Protein Hydrogel with Dynamic Schiff Base and Metal-Ligand Bonds for Diabetic Chronic Wound Recovery. Aggregate 2024, 5 (6), e639 10.1002/agt2.639.

[ref14] Liu Y.; Gong H.; Wang Z.; Yuan C.; Lu J.; Yan X. Treatment of Superbug Infection through a Membrane-Disruption and Immune-Regulation Cascade Effect Based on Supramolecular Peptide Hydrogels. Adv. Funct. Mater. 2023, 33 (45), 2305726 10.1002/adfm.202305726.

[ref15] Frederix P. W.; Scott G. G.; Abul-Haija Y. M.; Kalafatovic D.; Pappas C. G.; Javid N.; Hunt N. T.; Ulijn R. V.; Tuttle T. Exploring the Sequence Space for (Tri-) Peptide Self-Assembly to Design and Discover New Hydrogels. Nat. Chem. 2015, 7 (1), 30–37. 10.1038/nchem.2122. 25515887

[ref16] Tao K.; Levin A.; Adler-Abramovich L.; Gazit E. Fmoc-Modified Amino Acids and Short Peptides: Simple Bio-Inspired Building Blocks for the Fabrication of Functional Materials. Chem. Soc. Rev. 2016, 45 (14), 3935–3953. 10.1039/C5CS00889A. 27115033

[ref17] Chen C.; Wu D.; Wang Z.; Liu L.; He J.; Li J.; Chu B.; Wang S.; Yu B.; Liu W. Peptide-Based Hydrogel Scaffold Facilitates Articular Cartilage Damage Repair. ACS Appl. Mater. Interfaces 2024, 16 (9), 11336–11348. 10.1021/acsami.4c00811. 38407027

[ref18] Ahmadi Z.; Jha D.; Yadav S.; Singh A. P.; Singh V. P.; Gautam H. K.; Sharma A. K.; Kumar P. Self-Assembled Arginine–Glycine–Aspartic Acid Mimic Peptide Hydrogels as Multifunctional Biomaterials for Wound Healing. ACS Appl. Mater. Interfaces 2024, 16 (49), 67302–67320. 10.1021/acsami.4c14686. 39613718

[ref19] Holmes T. C.; de Lacalle S.; Su X.; Liu G.; Rich A.; Zhang S. Extensive Neurite Outgrowth and Active Synapse Formation on Self-Assembling Peptide Scaffolds. Proc. Natl. Acad. Sci. U.S.A. 2000, 97 (12), 6728–6733. 10.1073/pnas.97.12.6728. 10841570 PMC18719

[ref20] Panja S.; Seddon A.; Adams D. J. Controlling Hydrogel Properties by Tuning Non-Covalent Interactions in a Charge Complementary Multicomponent System. Chem. Sci. 2021, 12 (33), 11197–11203. 10.1039/D1SC02854E. 34522317 PMC8386653

[ref21] Guo Z.; Hou Y.; Tian Y.; Tian J.; Hu J.; Zhang Y. Antimicrobial Peptide Hydrogel with Ph-Responsive and Controllable Drug Release Properties for the Efficient Treatment of Helicobacter Pylori Infection. ACS Appl. Mater. Interfaces 2024, 16 (39), 51981–51993. 10.1021/acsami.4c09185. 39292612

[ref22] Kim J.; Saravanakumar G.; Choi H. W.; Park D.; Kim W. J. A Platform for Nitric Oxide Delivery. J. Mater. Chem. B 2014, 2 (4), 341–356. 10.1039/C3TB21259A. 32261379

[ref23] Kim J.; Yung B. C.; Kim W. J.; Chen X. Combination of Nitric Oxide and Drug Delivery Systems: Tools for Overcoming Drug Resistance in Chemotherapy. J. Controlled Release 2017, 263, 223–230. 10.1016/j.jconrel.2016.12.026. PMC5484762 28034787

[ref24] Kim T.; Nah Y.; Kim J.; Lee S.; Kim W. J. Nitric-Oxide-Modulatory Materials for Biomedical Applications. Acc. Chem. Res. 2022, 55 (17), 2384–2396. 10.1021/acs.accounts.2c00159. 35786846

[ref25] McCarthy C. W.; Goldman J.; Frost M. C. Synthesis and Characterization of the Novel Nitric Oxide (No) Donating Compound, S-Nitroso-N-Acetyl-D-Penicillamine Derivatized Cyclam (Snap-Cyclam). ACS Appl. Mater. Interfaces 2016, 8 (9), 5898–5905. 10.1021/acsami.5b12548. 26859235

[ref26] Silva R.; Fabry B.; Boccaccini A. R. Fibrous Protein-Based Hydrogels for Cell Encapsulation. Biomaterials 2014, 35 (25), 6727–6738. 10.1016/j.biomaterials.2014.04.078. 24836951

[ref27] Tabish T. A.; Crabtree M. J.; Townley H. E.; Winyard P. G.; Lygate C. A. Nitric Oxide Releasing Nanomaterials for Cardiovascular Applications. J. Am. Coll. Cardiol. 2024, 9 (5), 691–709. 10.1016/j.jacbts.2023.07.017. PMC11228123 38984042

[ref28] Hall C. N.; Garthwaite J. What Is the Real Physiological No Concentration in Vivo?. Nitric Oxide 2009, 21 (2), 92–103. 10.1016/j.niox.2009.07.002. 19602444 PMC2779337

[ref29] Mocellin S.; Bronte V.; Nitti D. Nitric Oxide, a Double Edged Sword in Cancer Biology: Searching for Therapeutic Opportunities. Med. Res. Rev. 2007, 27 (3), 317–352. 10.1002/med.20092. 16991100

[ref30] Qu J.; Zhao X.; Liang Y.; Xu Y.; Ma P. X.; Guo B. Degradable Conductive Injectable Hydrogels as Novel Antibacterial, Anti-Oxidant Wound Dressings for Wound Healing. Chem. Eng. J. 2019, 362, 548–560. 10.1016/j.cej.2019.01.028.

[ref31] Yang S.; Wang N.; Ouyang X. k.; Wu Y.; Hu J. A Novel No-Releasing Composite Hydrogel for Infected Wound Healing. Mater. Today Commun. 2024, 39, 109321 10.1016/j.mtcomm.2024.109321.

[ref32] Joseph C. A.; McCarthy C. W.; Tyo A. G.; Hubbard K. R.; Fisher H. C.; Altscheffel J. A.; He W.; Pinnaratip R.; Liu Y.; Lee B. P.; et al. Development of an Injectable Nitric Oxide Releasing Poly (Ethylene) Glycol-Fibrin Adhesive Hydrogel. ACS Biomater. Sci. Eng. 2018, 5 (2), 959–969. 10.1021/acsbiomaterials.8b01331. 31650030 PMC6812534

[ref33] Wang Y.; Yang X.; Chen X.; Wang X.; Wang Y.; Wang H.; Chen Z.; Cao D.; Yu L.; Ding J. Sustained Release of Nitric Oxide and Cascade Generation of Reactive Nitrogen/Oxygen Species Via an Injectable Hydrogel for Tumor Synergistic Therapy. Adv. Funct. Mater. 2022, 32 (36), 2206554 10.1002/adfm.202206554.

[ref34] Shishido S. M.; Seabra A. B.; Loh W.; Ganzarolli de Oliveira M. Thermal and Photochemical Nitric Oxide Release from S-Nitrosothiols Incorporated in Pluronic F127 Gel: Potential Uses for Local and Controlled Nitric Oxide Release. Biomaterials 2003, 24 (20), 3543–3553. 10.1016/s0142-9612(03)00153-4. 12809783

[ref35] Nie Y.; Zhang K.; Zhang S.; Wang D.; Han Z.; Che Y.; Kong D.; Zhao Q.; Han Z.; He Z.-X.; et al. Nitric Oxide Releasing Hydrogel Promotes Endothelial Differentiation of Mouse Embryonic Stem Cells. Acta Biomater. 2017, 63, 190–199. 10.1016/j.actbio.2017.08.037. 28859902

[ref36] Ma C. J.; He Y.; Jin X.; Zhang Y.; Zhang X.; Li Y.; Xu M.; Liu K.; Yao Y.; Lu F. Light-Regulated Nitric Oxide Release from Hydrogel-Forming Microneedles Integrated with Graphene Oxide for Biofilm-Infected-Wound Healing. Biomater. Adv. 2022, 134, 112555 10.1016/j.msec.2021.112555. 35523645

[ref37] Yang T.; Zelikin A. N.; Chandrawati R. Progress and Promise of Nitric Oxide-Releasing Platforms. Adv. Sci. 2018, 5 (6), 1701043 10.1002/advs.201701043. PMC6010811 29938181

[ref38] Shabbir M.; Atiq A.; Wang J.; Atiq M.; Saeed N.; Yildiz I.; Yan X.; Xing R.; Abbas M. Metal-Coordinated Amino Acid/Peptide/Protein-Based Supramolecular Self-Assembled Nanomaterials for Anticancer Applications. Aggregate 2025, 6 (1), e672 10.1002/agt2.672.

[ref39] Tasleem M.; Matouk A. M.; Abbas M. Design of Short Peptides for the Reduction of Silver Ions and Stabilization of Nanocomposites in Combating Bacterial Infections. ChemBioChem 2025, 26 (9), 2500122 10.1002/cbic.202500122. 40183352

[ref40] Tabish T. A.; Xu J.; Campbell C. K.; Abbas M.; Myers W. K.; Didwal P.; Carugo D.; Xie F.; Crabtree M. J.; Stride E.; et al. Ph-Sensitive Release of Nitric Oxide Gas Using Peptide-Graphene Co-Assembled Hybrid Nanosheets. Nitric Oxide 2024, 147, 42–50. 10.1016/j.niox.2024.04.008. 38631610 PMC7618039

[ref41] Abbas M.; Lipiński W. P.; Nakashima K. K.; Huck W. T.; Spruijt E. A Short Peptide Synthon for Liquid–Liquid Phase Separation. Nat. Chem. 2021, 13 (11), 1046–1054. 10.1038/s41557-021-00788-x. 34645986

[ref42] Yuan C.; Li Q.; Xing R.; Li J.; Yan X. Peptide Self-Assembly through Liquid-Liquid Phase Separation. Chem. 2023, 9 (9), 2425–2445. 10.1016/j.chempr.2023.05.009.

[ref43] Abbas M.; Law J. O.; Grellscheid S. N.; Huck W. T.; Spruijt E. Peptide-Based Coacervate-Core Vesicles with Semipermeable Membranes. Adv. Mater. 2022, 34 (34), 2202913 10.1002/adma.202202913. 35796384

[ref44] Patel A.; Lee H. O.; Jawerth L.; Maharana S.; Jahnel M.; Hein M. Y.; Stoynov S.; Mahamid J.; Saha S.; Franzmann T. M.; et al. A Liquid-to-Solid Phase Transition of the Als Protein Fus Accelerated by Disease Mutation. Cell 2015, 162 (5), 1066–1077. 10.1016/j.cell.2015.07.047. 26317470

[ref45] Zhou P.; Xing R.; Li Q.; Li J.; Yuan C.; Yan X. Steering Phase-Separated Droplets to Control Fibrillar Network Evolution of Supramolecular Peptide Hydrogels. Matter 2023, 6 (6), 1945–1963. 10.1016/j.matt.2023.03.029.

[ref46] Yuan C.; Xing R.; Cui J.; Fan W.; Li J.; Yan X. Multistep Desolvation as a Fundamental Principle Governing Peptide Self-Assembly through Liquid–Liquid Phase Separation. CCS Chem. 2024, 6 (1), 255–265. 10.31635/ccschem.023.202302990.

[ref47] Della Vecchia N. F.; Luchini A.; Napolitano A.; D’Errico G.; Vitiello G.; Szekely N.; d’Ischia M.; Paduano L. Tris Buffer Modulates Polydopamine Growth, Aggregation, and Paramagnetic Properties. Langmuir 2014, 30 (32), 9811–9818. 10.1021/la501560z. 25066905

[ref48] Liu J.; He X.; Zhang J. Z.; Qi L.-W. Hydrogen-Bond Structure Dynamics in Bulk Water: Insights from Ab Initio Simulations with Coupled Cluster Theory. Chem. Sci. 2018, 9 (8), 2065–2073. 10.1039/C7SC04205A. 29675248 PMC5885775

[ref49] Hua L.; Zhou R.; Thirumalai D.; Berne B. Urea Denaturation by Stronger Dispersion Interactions with Proteins Than Water Implies a 2-Stage Unfolding. Proc. Natl. Acad. Sci. U.S.A. 2008, 105 (44), 16928–16933. 10.1073/pnas.0808427105. 18957546 PMC2579355

[ref50] Kong J.; Yu S. Fourier Transform Infrared Spectroscopic Analysis of Protein Secondary Structures. Acta Biochim. Biophys. Sin. 2007, 39 (8), 549–559. 10.1111/j.1745-7270.2007.00320.x. 17687489

[ref51] Zou Y.; Li Y.; Hao W.; Hu X.; Ma G. Parallel Β-Sheet Fibril and Antiparallel Β-Sheet Oligomer: New Insights into Amyloid Formation of Hen Egg White Lysozyme under Heat and Acidic Condition from FTIR Spectroscopy. J. Phys. Chem. B 2013, 117 (15), 4003–4013. 10.1021/jp4003559. 23537140

[ref52] Sarma S.; Sudarshan T. R.; Nguyen V.; Robang A. S.; Xiao X.; Le J. V.; Helmicki M. E.; Paravastu A. K.; Hall C. K. Design of parallel β-sheet nanofibrils using Monte Carlo search, coarse-grained simulations, and experimental testing. Protein Sci. 2024, 33 (8), e5102 10.1002/pro.5102. 39037281 PMC11261811

[ref53] Yuan C.; Fan W.; Zhou P.; Xing R.; Cao S.; Yan X. High-Entropy Non-Covalent Cyclic Peptide Glass. Nat. Nanotechnol. 2024, 19 (12), 1840–1848. 10.1038/s41565-024-01766-3. 39187585

[ref54] Yang H.; Yang S.; Kong J.; Dong A.; Yu S. Obtaining Information About Protein Secondary Structures in Aqueous Solution Using Fourier Transform IR Spectroscopy. Nat. Protoc. 2015, 10 (3), 382–396. 10.1038/nprot.2015.024. 25654756

[ref55] Micsonai A.; Wien F.; Kernya L.; Lee Y.-H.; Goto Y.; Réfrégiers M.; Kardos J. Accurate Secondary Structure Prediction and Fold Recognition for Circular Dichroism Spectroscopy. Proc. Natl. Acad. Sci. U.S.A. 2015, 112 (24), E3095–E3103. 10.1073/pnas.1500851112. 26038575 PMC4475991

[ref56] Zou Q.; Chang R.; Xing R.; Yuan C.; Yan X. Injectable Self-Assembled Bola-Dipeptide Hydrogels for Sustained Photodynamic Prodrug Delivery and Enhanced Tumor Therapy. J. Controlled Release 2020, 319, 344–351. 10.1016/j.jconrel.2020.01.002. 31917297

[ref57] Lima-Sousa R.; Alves C. G.; Melo B. L.; Costa F. J.; Nave M.; Moreira A. F.; Mendonça A. G.; Correia I. J.; de Melo-Diogo D. Injectable Hydrogels for the Delivery of Nanomaterials for Cancer Combinatorial Photothermal Therapy. Biomater. Sci. 2023, 11 (18), 6082–6108. 10.1039/D3BM00845B. 37539702

[ref58] Thomas D. D. Breathing New Life into Nitric Oxide Signaling: A Brief Overview of the Interplay between Oxygen and Nitric Oxide. Redox Biol. 2015, 5, 225–233. 10.1016/j.redox.2015.05.002. 26056766 PMC4473092

[ref59] Moilanen E.; Vapaatalo H. Nitric Oxide in Inflammation and Immune Response. Ann. Med. 1995, 27 (3), 359–367. 10.3109/07853899509002589. 7546626

[ref60] Andrabi S. M.; Sharma N. S.; Karan A.; Shahriar S. M. S.; Cordon B.; Ma B.; Xie J. Nitric Oxide: Physiological Functions, Delivery, and Biomedical Applications. Adv. Sci. 2023, 10 (30), 2303259 10.1002/advs.202303259. PMC10602574 37632708

[ref61] Kreuger J.; Phillipson M. Targeting Vascular and Leukocyte Communication in Angiogenesis, Inflammation and Fibrosis. Nat. Rev. Drug Discovery 2016, 15 (2), 125–142. 10.1038/nrd.2015.2. 26612664

