# Supplementary material for: Self-Assembled Peptide-Based Fibrous Hydrogel as a Biological Catalytic Scaffold for Nitric Oxide Generation and Encapsulation
Source: ACS Appl Mater Interfaces. 2025 Apr 29;17(19):27964–73. doi: 10.1021/acsami.5c03250 (PMC12086768; doi:10.1021/acsami.5c03250)
Supplement: Supplementary file 1 — am5c03250_si_001.pdf [file am5c03250_si_001.pdf]

Supporting information

For

Self-Assembled Peptide-Based Fibrous Hydrogel as Biological Catalytic Scaffold for Nitric Oxide Generation and Encapsulation

*Muhammad Younis[a]#, Tanveer A. Tabish[b]#, Cherly Firdharini[a], Mohamed Aslam[a], Mostafa Khair[c], Dalaver H. Anjum[d], Xuehai Yan[e, f], and Manzar Abbas[a, g]**

[a]Department of Chemistry, Khalifa University of Science and Technology, P.O. Box 127788, Abu Dhabi, United Arab Emirates

[b]Division of Cardiovascular Medicine, Radcliffe Department of Medicine, British Heart Foundation (BHF) Centre of Research Excellence, University of Oxford, Oxford, OX3 7BN, United Kingdom

[c]Core Technology Platforms, New York University Abu Dhabi, Abu Dhabi, United Arab Emirates

[d]Department of Physics, Khalifa University of Science and Technology, P.O. Box 127788, Abu Dhabi, United Arab Emirates

[e]University of Chinese Academy of Sciences, Beijing 100049, P.R. China

[f]State Key Laboratory of Biochemical Engineering, Institute of Process Engineering, Chinese Academy of Sciences, Beijing 100190, P.R. China

[g]Functional Biomaterial Group, Khalifa University of Science and Technology, P.O. Box 127788, Abu Dhabi, United Arab Emirates

Correspondence to: Manzar Abbas (manzar.abbas@ku.ac.ae)

1. Synthesis of peptide derivatives 3
2. Characterization of peptide derivatives 4
2.1. Purification 4
2.2. LC-MS analysis 4
2.3. Nuclear Magnetic Resonance (NMR) 4
3. Spectroscopic experiments 10
3.1. Fourier Transform Infrared Spectroscopy (FT-IR) 10
3.2. Circular Dichroism Spectroscopy 10
4. Mechanical properties 10
5. Powder X-ray Diffraction (XRD) 10
6. Computational details 11
7. Inflammatory responses 11
8. Supplementary figures 12
9. References 25

1. Synthesis of peptide derivatives

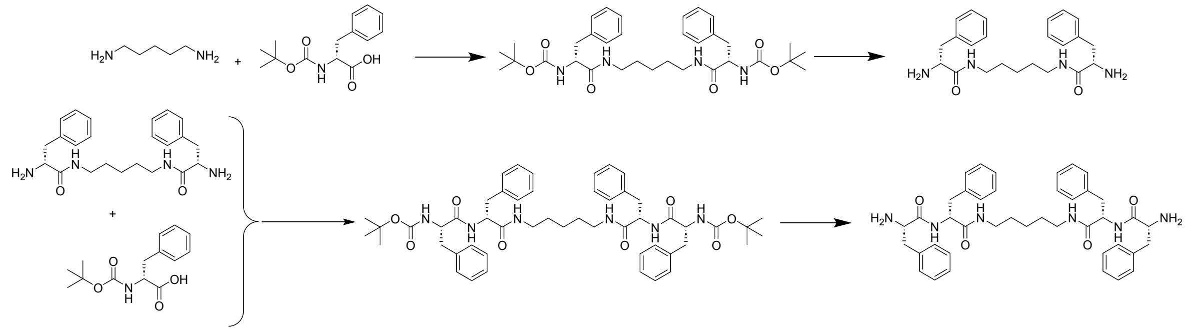

Step 1. Synthesis of Boc-Fc$_5$F-Boc

The reactant Boc-Phe-OH 500 mg (1.9 mmol), coupling reagents HATU 720 mg (1.86 mmol), and HOBT 250 mg (1.86 mmol) were dissolved in 3 mL anhydrous dimethylformamide (DMF). Under continuous stirring, the base DIPEA 750 µL (5.8 mmol, three equivalents) was added, and subsequently, the linker cadaverine (c$_5$) 117 µL (1.0 mmol) was added. The reaction mixture was stirred for 24 hours at room temperature. After 24 hours, the reaction mixture was poured into 20 mL of water to collect the white precipitate through filtration. It was dried under a vacuum after washing it with plenty of water.

Step 2. Deprotection of Boc-Fc$_5$F-Boc

The product from the first step (Boc-Fc$_5$F-Boc) (304.31 mg, 0.51 mmol) was dissolved in 4:1 dioxane/DCM (1.5 mL) in a round-bottom flask. To deprotect the intermediate product, 4 M hydrogen chloride solution in dioxane (3 mL) was added, and the reaction mixture was stirred for three hours. The solvent was evaporated on a rotary evaporator, yielding oily residue. Diethyl ether (40 mL) was added to the flask, and the content was gently stirred. The white precipitate was separated by centrifugation. LC-MS was used to characterize the product.

Step 3. Synthesis of Boc-FFc$_5$FF-Boc

The reactant Boc-Phe-OH 500 mg (1.9 mmol), coupling reagents HATU 720 mg (1.86 mmol), and HOBT 250 mg (1.86 mmol) were dissolved in 3 mL anhydrous dimethylformamide (DMF). Under continuous stirring, the base DIPEA 750 µL (5.8 mmol, three equivalents) was added, and subsequently, the linker Fc$_5$F (356.62 mg, 0.9 mmol) was added. The reaction mixture was stirred for 24 hours at room temperature. After 24 hours, the reaction mixture was poured into 20 mL of water to collect the white precipitates through filtration. It was dried under a vacuum after washing it with plenty of water.

Step 4. Synthesis of FFc$_5$FF

The product from the third step (Boc-FFc$_5$FF-Boc) (454.15 mg, 0.51 mmol) was dissolved in dioxane/DCM (1.5 mL) in a round-bottom flask. 4 M hydrogen chloride solution in dioxane (3 mL) was added for deprotection, and the reaction mixture was stirred for three hours. The solvent was evaporated on a rotary evaporator, yielding an oily residue. Diethyl ether (40 mL) was added to the flask, and the content was gently stirred. A white precipitate formed and was 4 separated by centrifugation. The final product was obtained after freeze-drying.

2. Characterization of peptide derivatives

2.1. Purification

All synthesized products were purified using prep-HPLC (Thermo Scientific Ultimate 3000) using an Agilent InfinityLab ZORBAX 5 SB-C18 column (250 × 21.2 mm; particle size: 5 µm; pore size 80 Å) on an ultimate 3000 system. A gradient method 5-100 % of phase B in phase A during 26 minutes (phase A: 100 % H_2O with 0.1% trifluoracetic acid (TFA), phase B: 100% MeCN with 0.1% TFA) and flow rate 20.0 mL/min was used with UV detection at wavelength 220 nm and 254 nm.

Time	% B
0.000	5.0
25.000	80.0
26.000	100.0
29.000	100.0
30.000	5.0
32.000	5.0

2.2. LC-MS analysis

After purification of all synthesized compounds, we used analytical HPLC (Thermo Scientific Ultimate 3000) coupled with a Q Exactive Orbitrap mass spectrometer to test the liquid chromatogram and mass spectra of each compound. Agilent ZORBAX Eclipse Plus C18 column (4.6 x 150 mm, 5 µm) was used with a gradient method 5-90 % of phase B in phase A for 26 minutes (phase A: 100 % H_2O with 0.1% trifluoracetic acid (TFA), phase B: 100% MeCN with 0.1% TFA). The flow rate was 0.5 mL/min, and a UV detector at wavelengths 220 nm and 254 nm was used. Full scan mode with a heated ESI source and positive polarity was used for mass spectra. The column temperature was maintained at 35 ^oC.

2.3. Nuclear Magnetic Resonance (NMR)

^1H and ^{13}CNMR of synthesized compounds were performed in DMSO-d*6* using Bruker, 500 MHz. Briefly, 3 mg of each compound was dissolved in 500 µL DMSO-d6.

FFc$_5$FF

^1HNMR (500 MHz, DMSO) δ 8.80 (d, *J* = 8.2 Hz, 2H), 8.06 (s, 4H), 8.01 (t, *J* = 5.6 Hz, 2H), 7.24 – 7.16 (m, 18H), 7.16 – 7.12 (m, 2H), 4.43 (td, *J* = 8.0, 6.5 Hz, 2H), 3.97 (dd, *J* = 7.6, 5.4 Hz, 2H), 3.04 (dd, *J* = 14.1, 5.3 Hz, 2H), 2.95 (dt, *J* = 13.2, 6.6 Hz, 2H), 2.88 (dd, *J* = 13.8, 7.1 Hz, 5H), 2.80 (dd, *J* = 13.7, 8.0 Hz, 2H), 1.20 (ddd, *J* = 18.4, 9.5, 3.7 Hz, 5H), 1.02 – 0.97 (m, 2H). ^{13}CNMR (126 MHz, DMSO) δ 170.31, 168.15, 137.81, 135.22, 130.09, 129.68, 128.90, 128.63, 127.55, 126.91, 54.93, 53.60, 38.90, 38.60, 37.35, 29.11, 24.05.

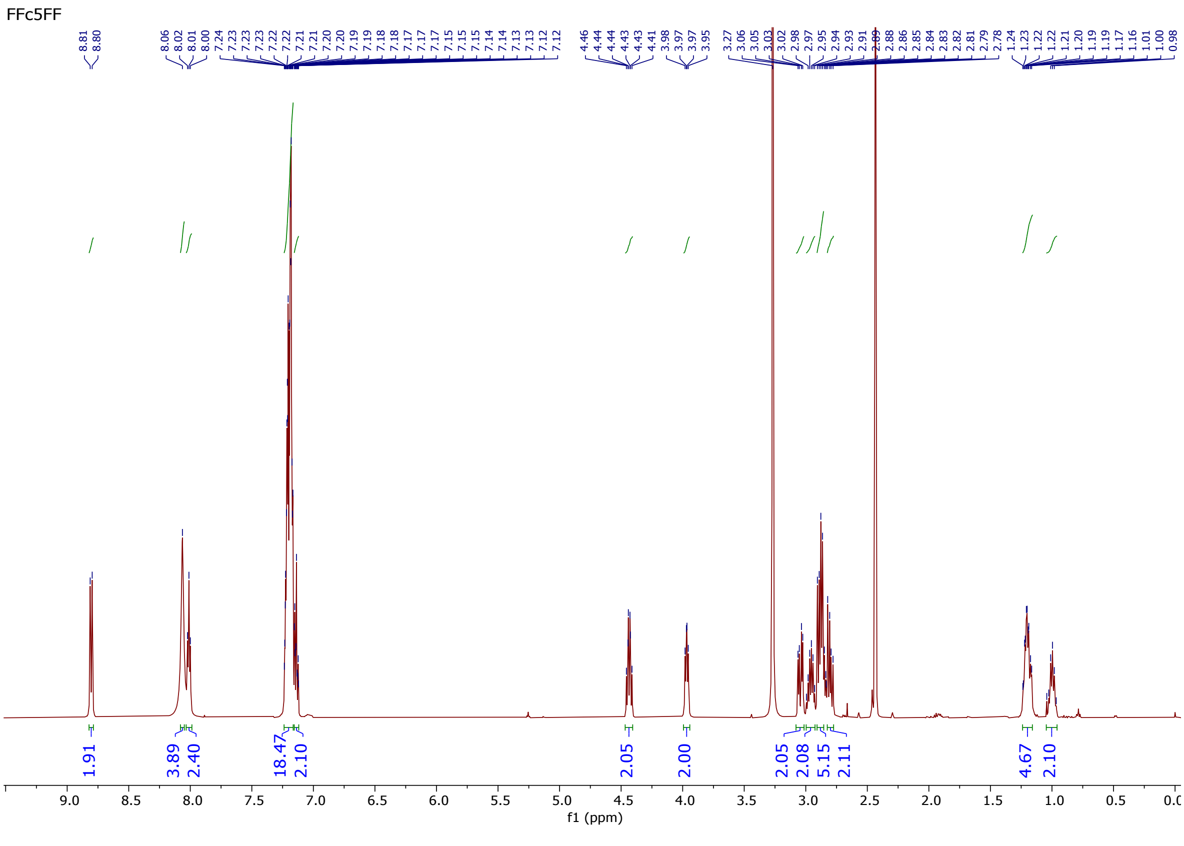
FFc5FF
f1 (ppm)

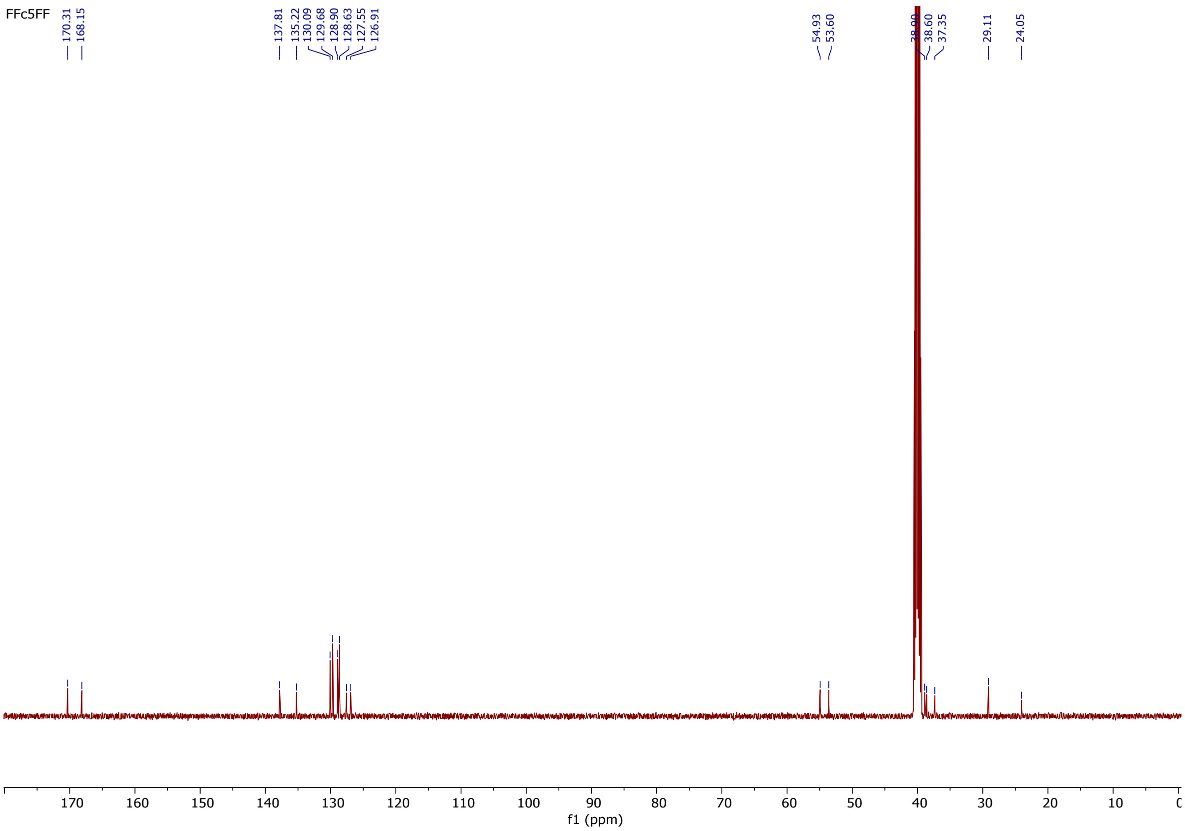

^{1}HNMR spectrum (top) and ^{13}CNMR spectrum (bottom) of FFc_5FF in DMSO-d6.

LFc_5FL

^{1}HNMR (500 MHz, DMSO) δ 8.78 (d, *J* = 8.2 Hz, 2H), 8.09 (s, 4H), 8.03 (t, *J* = 5.6 Hz, 2H), 7.34 – 7.12 (m, 10H), 4.51 (q, *J* = 7.7 Hz, 2H), 3.78 (s, 2H), 3.02 – 2.93 (m, 4H), 2.93 – 2.83 (m, 4H), 1.66 – 1.58 (m, 2H), 1.52 (dt, *J* = 8.0, 6.0 Hz, 2H), 1.23 (td, *J* = 7.1, 2.8 Hz, 4H), 1.06 – 0.98 (m, 2H), 0.88 (dd, *J* = 6.5, 4.5 Hz, 12H).^{13}CNMR (126 MHz, DMSO) δ 170.35, 169.22, 158.43, 158.18, 137.82, 129.61, 128.64, 126.90, 54.85, 51.14, 40.72, 38.83, 38.45, 29.06, 23.93, 23.89, 23.26, 22.20.

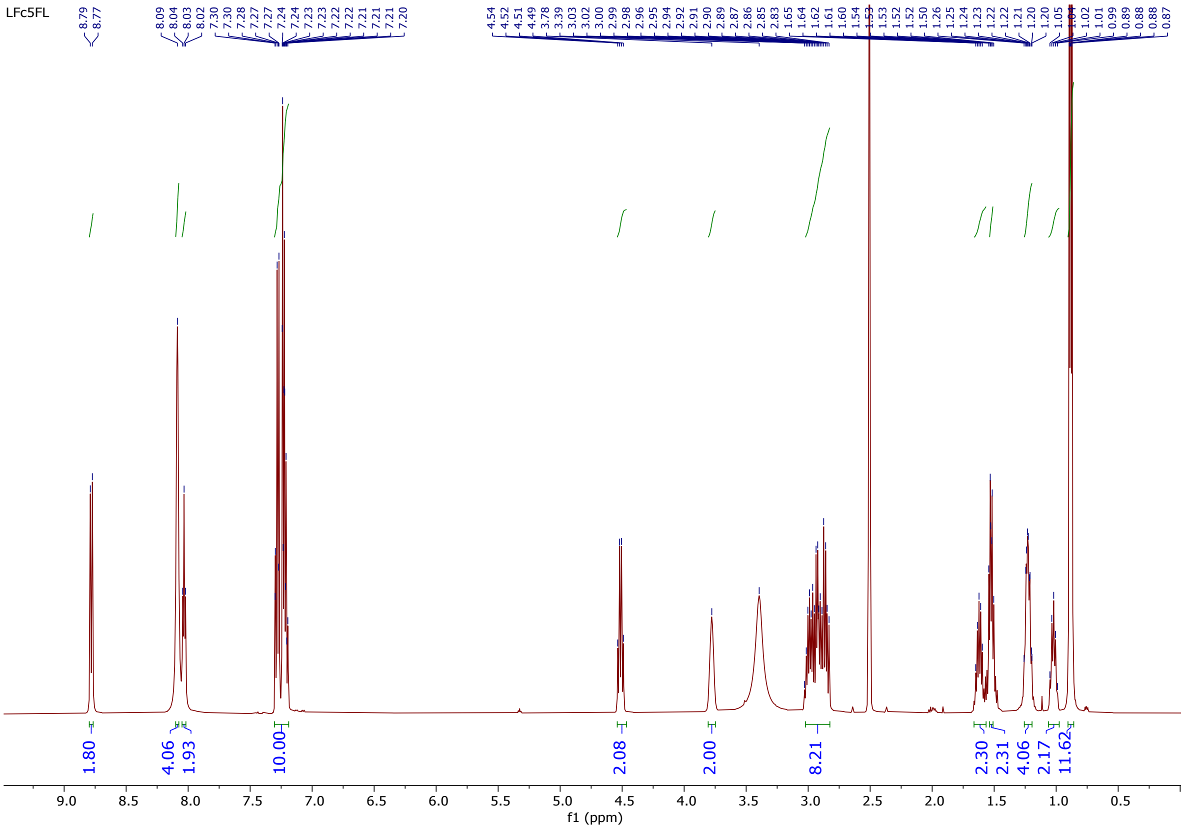
LFc5FL
1.80
4.06
1.93
10.00
2.08
2.00
8.21
2.30
2.31
4.06
2.17
11.62
9.0 8.5 8.0 7.5 7.0 6.5 6.0 5.5 5.0 4.5 4.0 3.5 3.0 2.5 2.0 1.5 1.0 0.5
f1 (ppm)

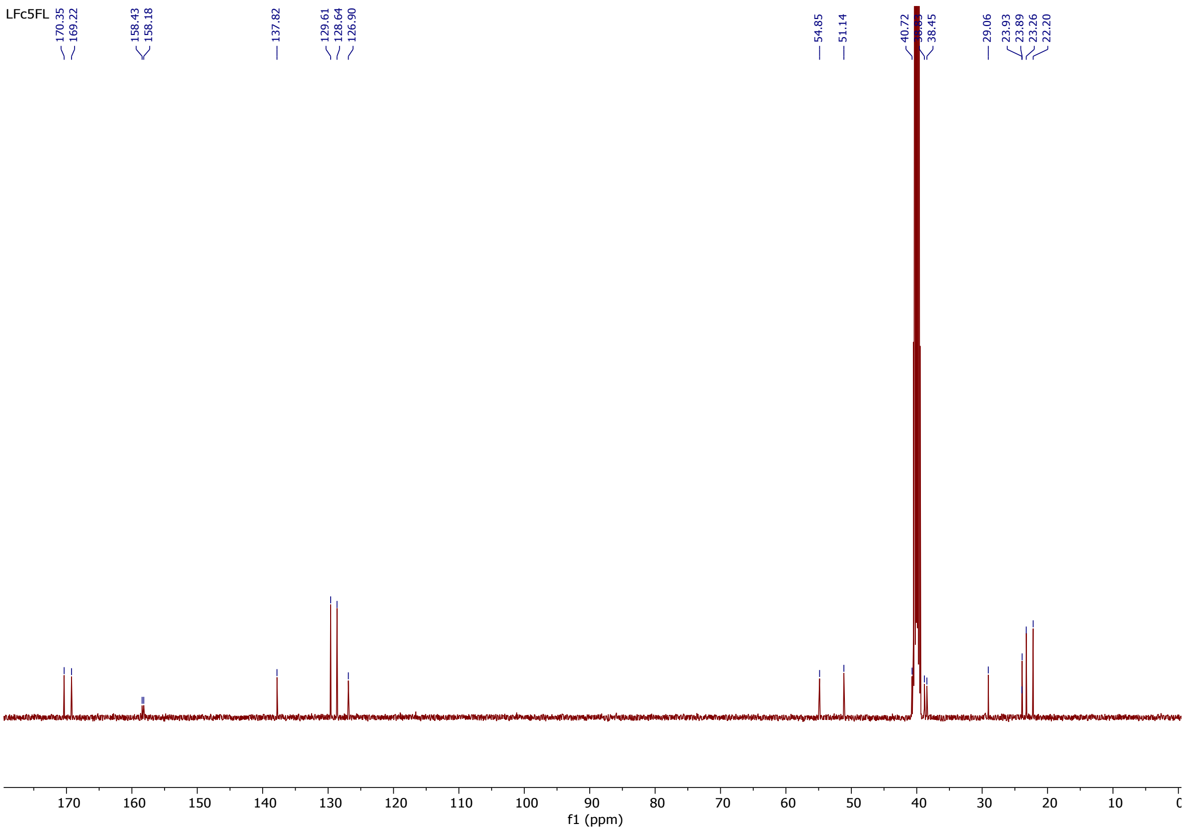

^{1}HNMR spectrum (top) and ^{13}CNMR spectrum (bottom) of LFc_5FL in DMSO-d6.

LLc_5LL

^{1}HNMR (500 MHz, DMSO) δ 8.58 (d, J = 8.3 Hz, 2H), 8.15 – 8.00 (m, 6H), 4.33 (td, J = 8.6, 6.2 Hz, 2H), 3.79 (s, 2H), 3.05 – 2.96 (m, 4H), 1.66 – 1.55 (m, 4H), 1.55 – 1.48 (m, 3H), 1.47 – 1.42 (m, 4H), 1.40 – 1.33 (m, 3H), 1.25 – 1.17 (m, 2H), 0.92 – 0.85 (m, 24H). ^{13}CNMR (126 MHz, DMSO) δ 171.47, 168.98, 158.43, 158.19, 118.95, 116.56, 51.70, 51.16, 41.79, 40.73, 38.81, 29.09, 24.55, 23.98, 23.93, 23.31, 23.15, 22.39.

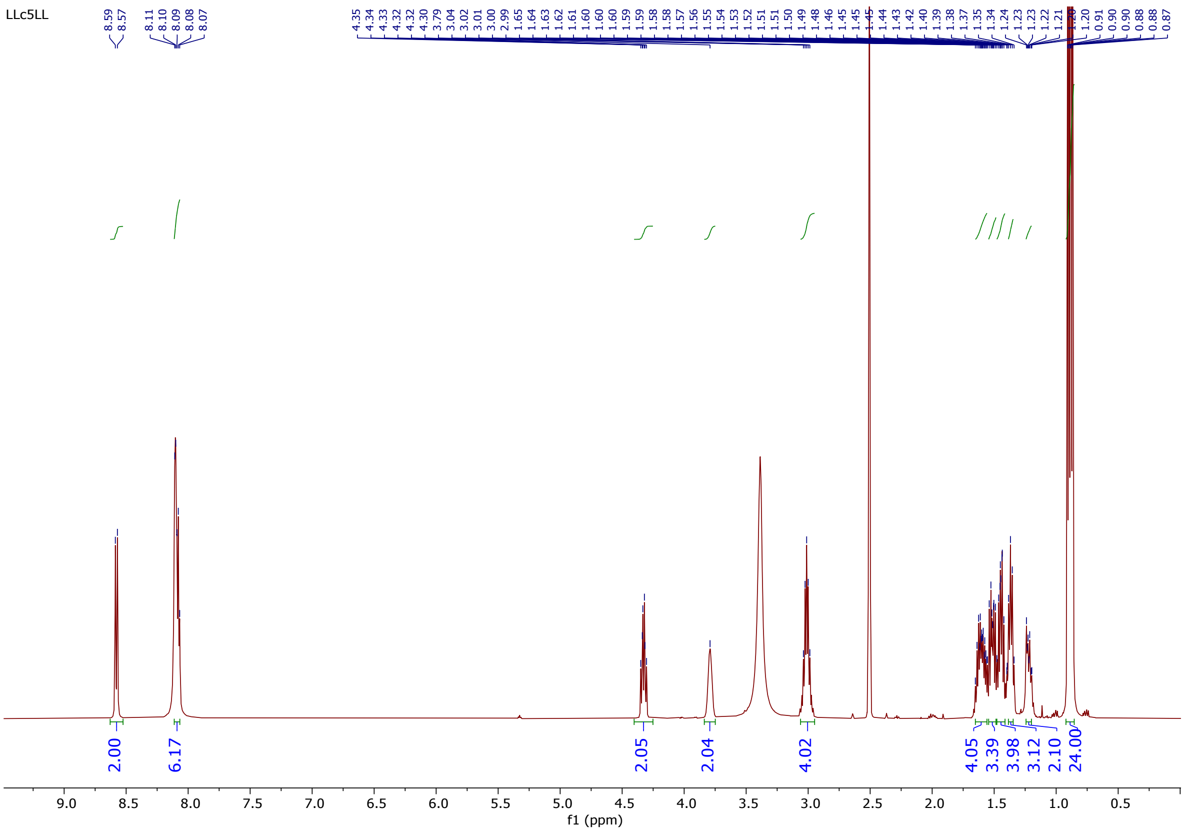
LLc5LL
2.00
6.17
2.05
2.04
4.02
4.05
3.39
3.98
3.12
2.10
24.00
f1 (ppm)

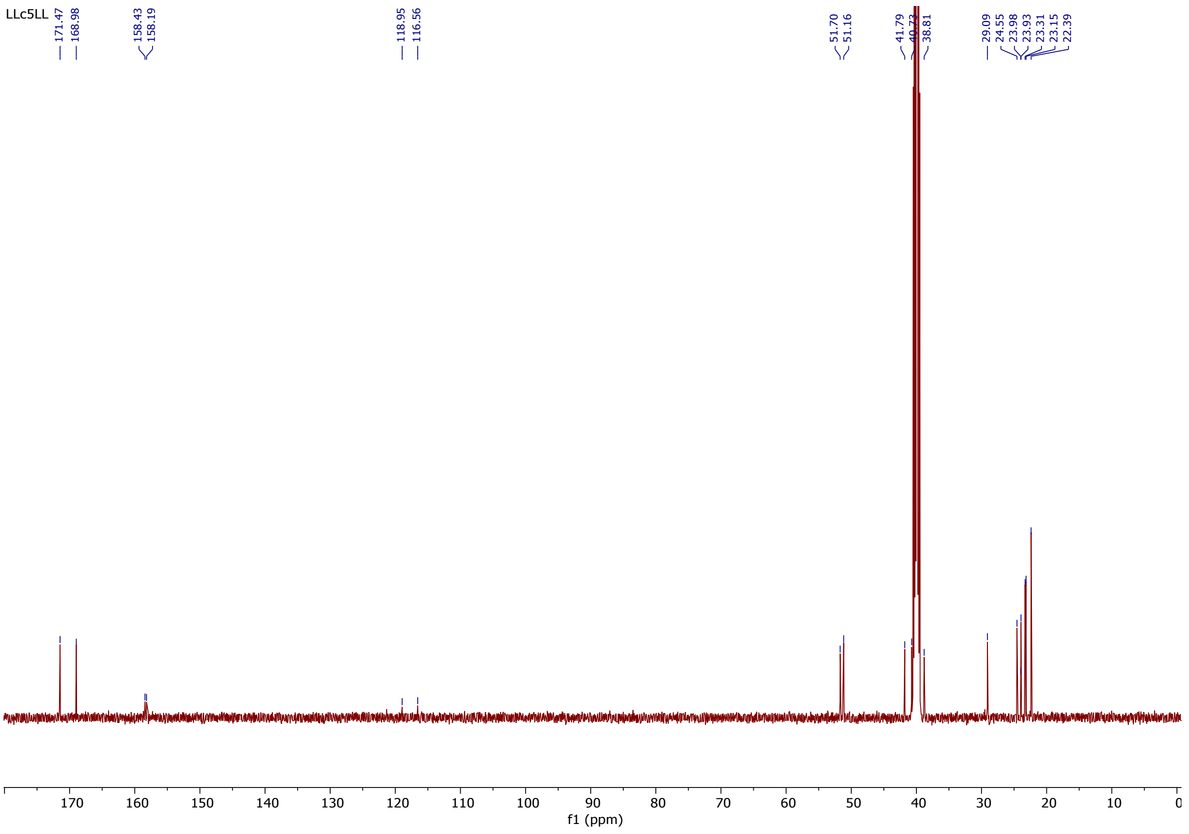

^{1}HNMR spectrum (top) and ^{13}CNMR spectrum (bottom) of LLc$_5$LL in DMSO-d6.

3. Spectroscopic Experiments

3.1. Fourier Transform Infrared Spectroscopy (FT-IR)

The hydrogels of FFc$_5$FF and LFc$_5$FL with different concentrations (1 - 10 mg/mL) were prepared and freeze-dried, and samples were observed using the FTIR (Thermo Scientific, Nicolet iS10, Smart iTR) in ATR mode from 500 to 4000 cm^{-1}.

3.2. Circular Dichroism Spectroscopy

CD spectra were collected between 190 and 260 nm at room temperature at a 1 nm/s scan rate in 1 mm quartz cuvettes using a Chirascan CD spectrometer (Applied Photophysics). CD spectra of the buffer alone or buffer containing different peptide hydrogel concentrations were examined for the secondary structural changes using 1.0 mg/mL and 5.0 mg/mL concentrations of FFc$_5$FF and LFc$_5$FL.

4. Mechanical Properties

The hydrogel samples of FFc$_5$FF and LFc$_5$FL (5 mg/mL) were prepared and aged for 2 hours before rheological analysis. After that, the samples were gently deposited in the middle of the parallel plates of the diameter of the rheometer (discovery hr-1 hybrid rheometer) at 23 ^{o}C. Dynamic oscillatory frequency sweep measurements were conducted at a 1% strain amplitude.

5. Powder X-ray Diffraction (XRD)

The lyophilized hydrogels of FFc$_5$FF (5.0 mg/mL), LFc$_5$FL (5.0 mg/mL), and powder form of both compounds with the same concentration were analyzed in the XRD pattern using an

X-ray diffractometer PANalytical Empyrean instrument. The spectra were recorded by Cu Kα radiation (λ = 1.54 A°) and a nickel monochromator filtering wave at 40 kV and 30 mA. The diffraction pattern was obtained at diffraction angles 2θ between 5° and 80°, with a scanning speed of 0.4°/min at room temperature.

6. Computational details

The protonation state of the FFc_5FF was adjusted at pH 7.4 using Avogadro v1.2.0. Then, the molecular structure was subjected to geometric optimization utilizing the RHF method and def2-SVP basis set using ORCA software v 5.0.4. For molecular dynamics simulation studies, the topology parameters of the compound were obtained by the ACPYPE server (https://www.bio2byte.be/acpype/). The force field was set to GAFF, suitable for a wide range of organic molecules, and the charge was set to 2. Using GROMACS 2024.2, 64 molecule copies were inserted into a cubic box and solvated with the TIP3P water model. The system was neutralized by adding Cl^- ions; then, energy was minimized in 50,000 steps by the steepest descent algorithm. The equilibration was performed in two phases. An NVT (canonical) ensemble was used in the first phase, and a V-rescale thermostat algorithm was employed. The system was heated from 500 ps to 300 K. The NPT (Isothermal-Isobaric) ensemble was used in the second phase, where the Berendsen algorithm was first employed. The pressure was kept constant at 1 bar for 500 ps, then for another 500 ps employing the C-rescale algorithm, and finally for another 500 ps using the Parrinello-Rahman algorithm, which allows for flexibility in the shape and size of the simulation box, thereby accurately reproducing the conditions of constant pressure[1]. The advantage of equilibrating the system using the NPT ensemble in three steps is that, in the first step, starting with the Berendsen algorithm, it helps avoid initial shocks to the system by soft-coupling to the desired temperature and pressure. Then, transitioning to the C-rescale algorithm improves the statistical accuracy of the temperature control, preparing the system for a more precise and physically representative pressure control. Finally, the simulation can accurately reflect volume fluctuations and molecular interactions under a controlled pressure environment. The H-bonds were constrained by using the Linear Constraint Solver (LINCS) algorithm. Finally, the MDS production was run for 200 ns under constant pressure (NPT ensemble), and long-range electrostatic interactions were computed using the Particle Mesh Ewald (PME) algorithm. The cutoff values for van der Waals and electrostatic interactions were 1.2 nm. Bond energies were calculated using ORCA software 5.0.4.

7. Inflammatory responses

Mouse RAW264.7 macrophage lineage cells (1×10^5 cells/ well, passage 2) were seeded on 24-well plates and allowed to grow in a complete medium at 37 °C. The culture medium was removed after 24 hours and washed with PBS. The cells were pretreated with lipopolysaccharide (LPS) (50 ng/mL) for 3 hours. The supernatants were collected by centrifugation. Cytokine concentrations released from macrophages were evaluated using ELISA. Briefly, the supernatant harvested after macrophages were cultured with NO encapsulated FFc_5FF hydrogels at different concentration for 24 hours was analyzed for the release of IL-6 and TNF-α using ELISA kits (RD systems, USA).

8. Supplementary figures

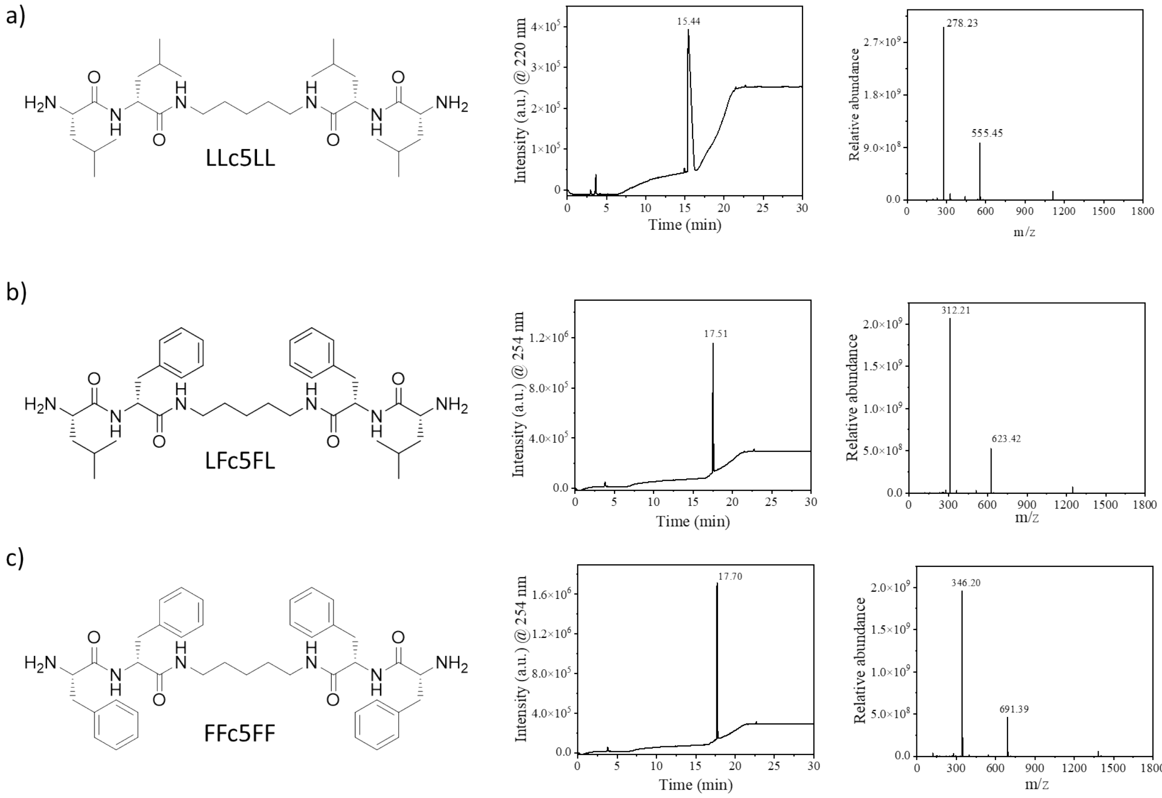

Figure S1. Chemical structures, liquid chromatogram, and mass spectra of designer peptide derivatives, a) LLc_5LL, b) LFc_5FL, and c) FFc_5FF.

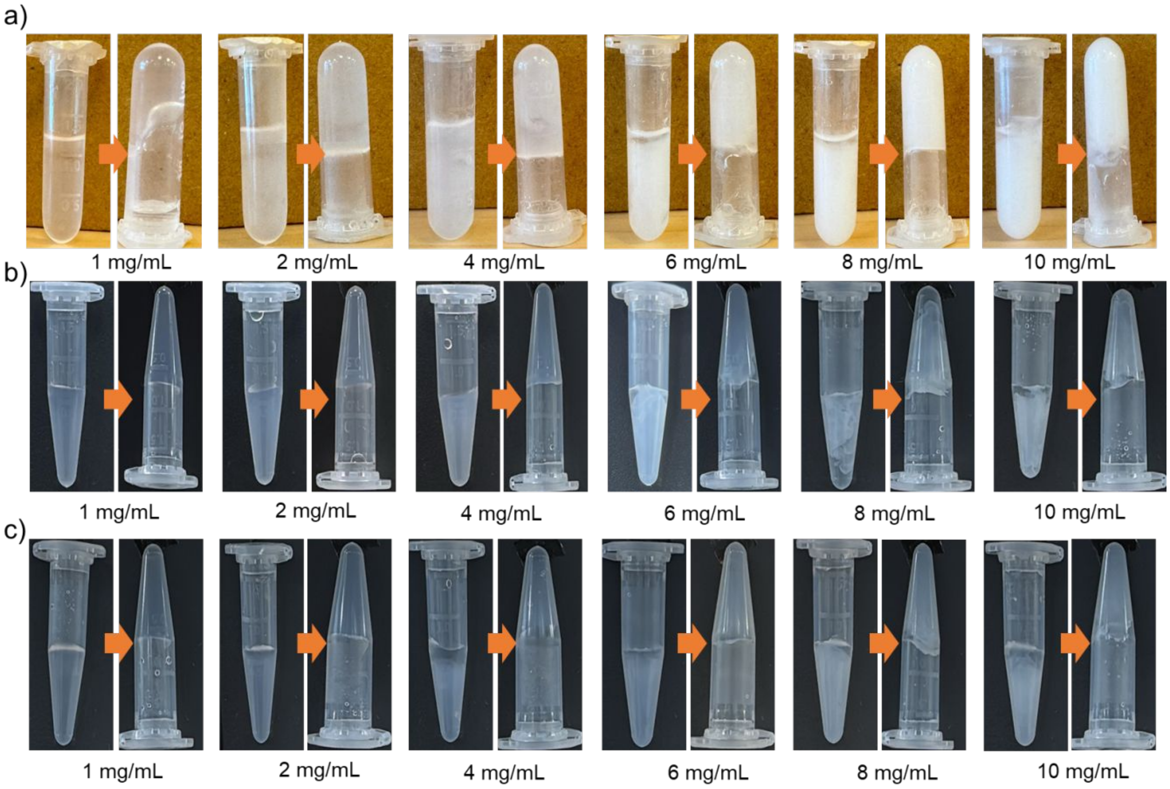

Figure S2. a) Gelation behavior of FFc_5FF at different concentrations in tris buffer (1M) – pH 8.0, b) gelation of FFc_5FF at different concentrations occurred in phosphate buffer (1M) –pH 7.4, c) gelation of FFc_5FF at different concentrations occurred in HEPES buffer (1M) – pH 7.5

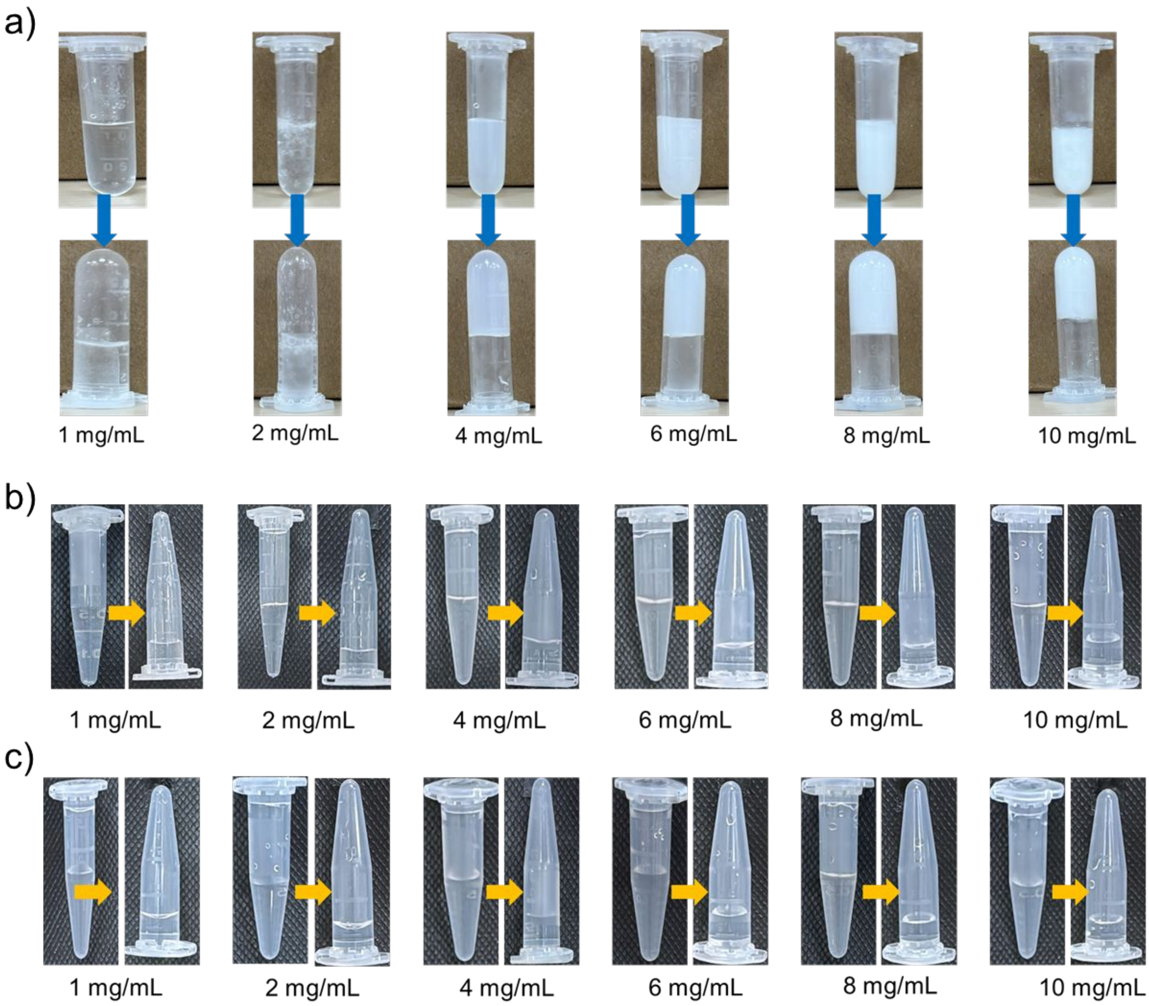

Figure S3. a) Gelation behavior of LFc_5FL at different concentrations in tris buffer (1M) –pH 8.0, b) No gelation of LFc_5FL at different concentrations occurred in phosphate buffer (1M) –pH 7.4, c) No gelation of LFc_5FL at different concentrations occurred in HEPES buffer (1M) –pH 7.5.

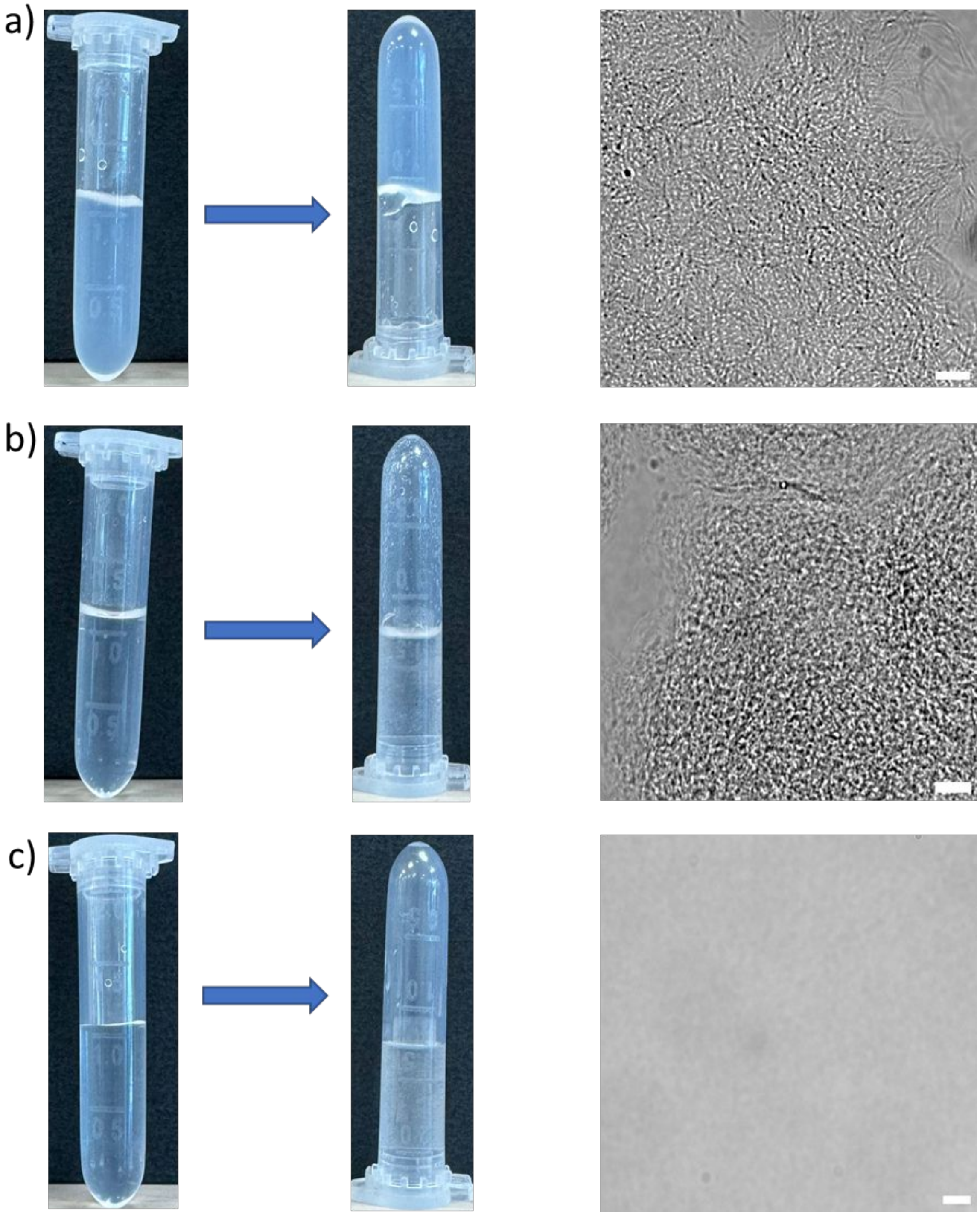

Figure S4. a) FFc$_5$FF, b) LFc$_5$FL, and c) LLc$_5$LL at 1 mg/mL concentration and gelation in tris- buffer 200 mM (pH 8.0). The scale bar is 20 μm.

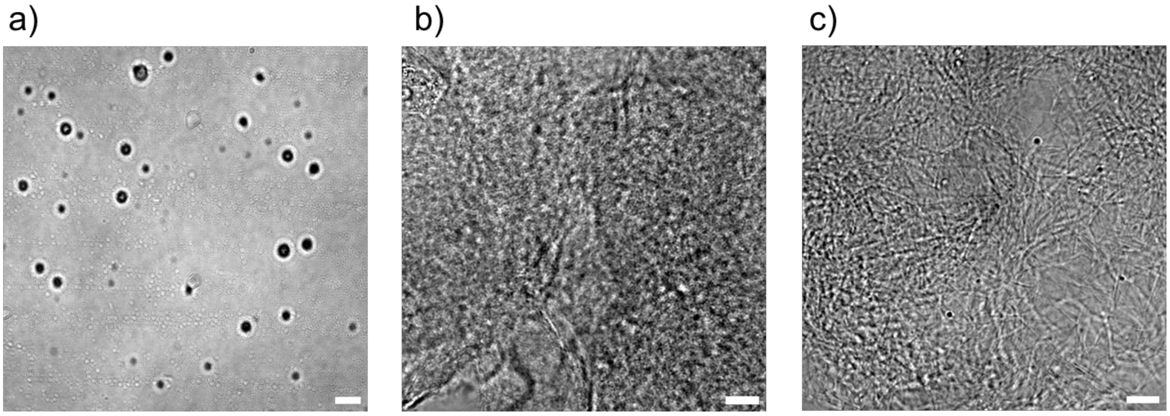

Figure S5. Optical microscope images of samples prepared in NaOH, a) LLc5LL (20 mg/mL) form liquid droplets, b) LFc_5FL(1mg/mL) aggregate, c) FFc_5FF (1 mg/mL) nanofibers. The scale bar is 20 µm.

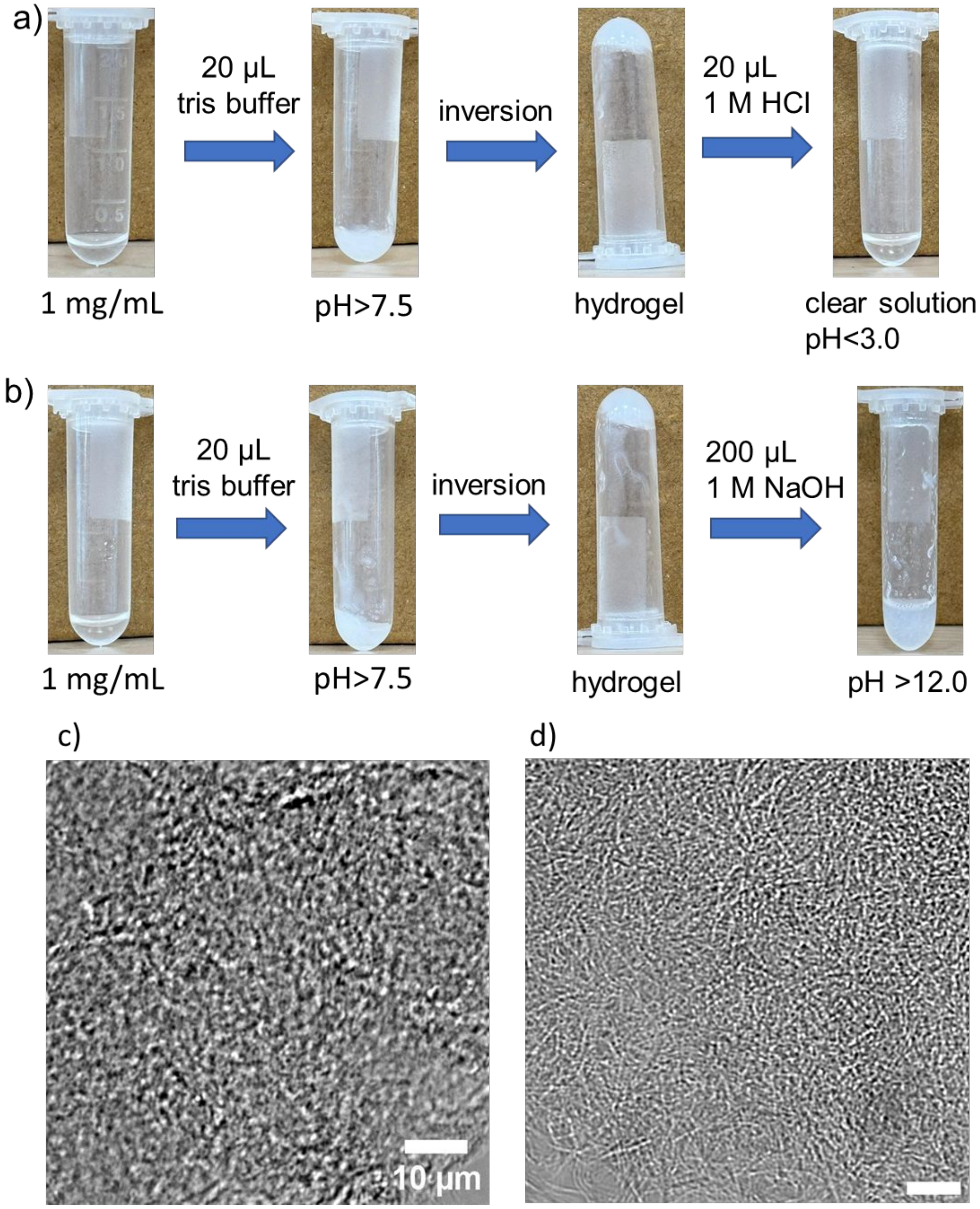

Figure S6. Responsive behavior of FFc5FF hydrogel, a) photographs of Eppendorf tubes containing hydrogel and its transformation into transparent solution, b) photographs of Eppendorf tubes containing hydrogel and its transformation into aggregation as shown in optical microscope image in c, d) optical microscope image after heating the hydrogel at 37°C for 2 hours. Scale bar in d is same.

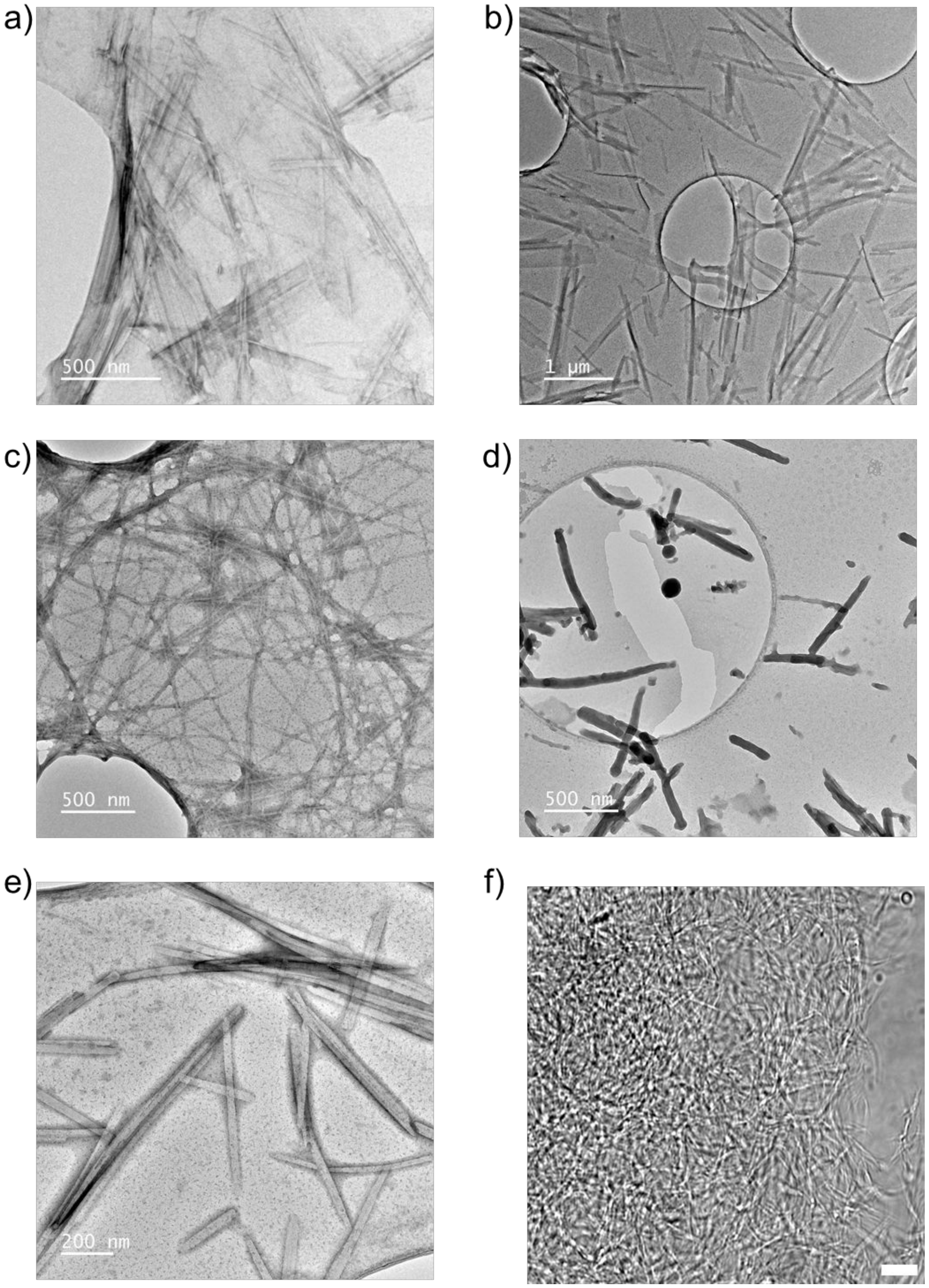

Figure S7. TEM images, a) FFc_5FF (1 mg/mL) hydrogel in tris buffer, b) FFc_5FF (5 mg/mL) hydrogel in tris buffer after 24 hours aging, c) LFc_5FL (1 mg/mL) hydrogel in tris buffer, d) LFc_5FL (5 mg/mL) hydrogel in tris buffer, e) LFc_5FL (5 mg/mL) hydrogel in tris buffer after 24 hours aging time, f) optical microscope image after adding 200 μL urea (3M) in LFc_5FL (5 mg/mL) hydrogel for two hours. scale bar is 10 μm.

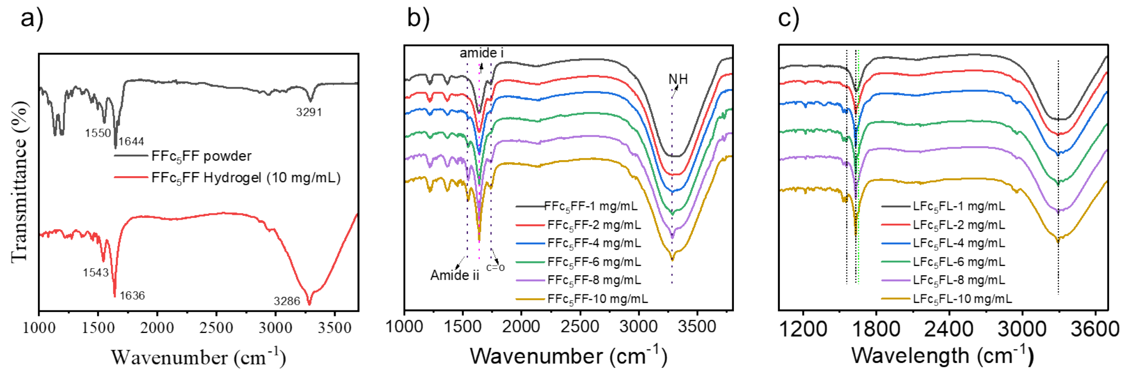

Figure S8. FTIR spectra, a) FFc_5FF lyophilized powder and hydrogel, b) FFc_5FF at different concentrations, c) LFc_5FL at different concentrations.

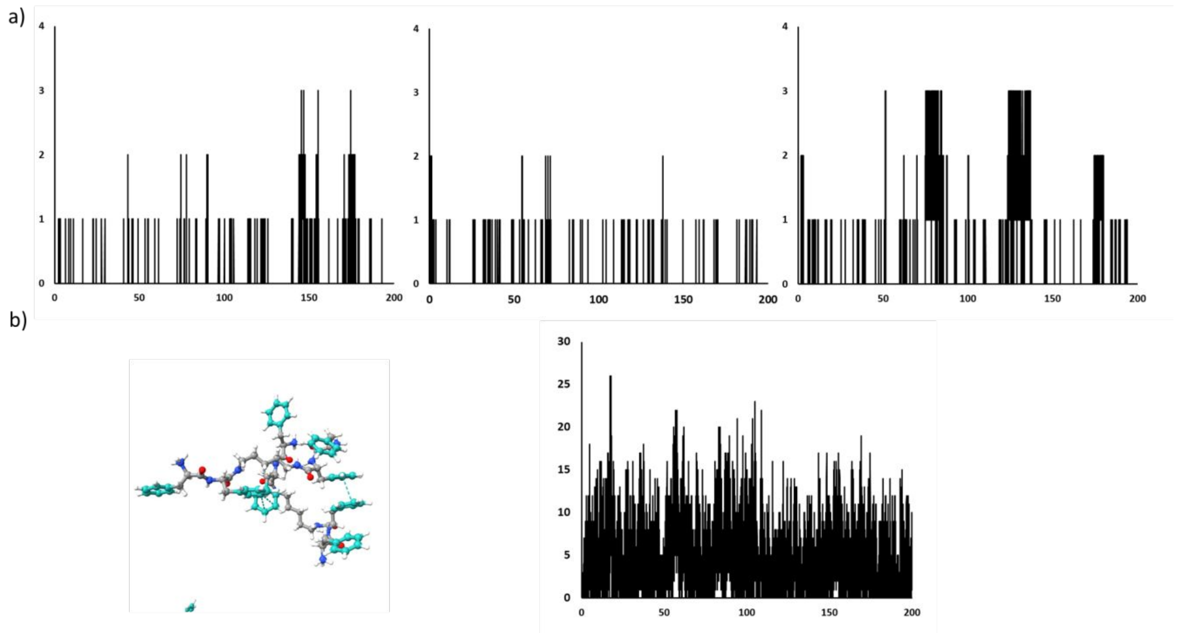

Figure S9. a) Total number of intramolecular hydrogen bonds formed during the simulation run for a random sample of the copies of the molecule. The x-axis represents time (ns), the y-axis represents the number of hydrogen bonds, and b) The total number of hydrophobic interactions formed between the compound molecules during the simulation. The x-axis represents time (ns), and the y-axis represents the number of interactions.

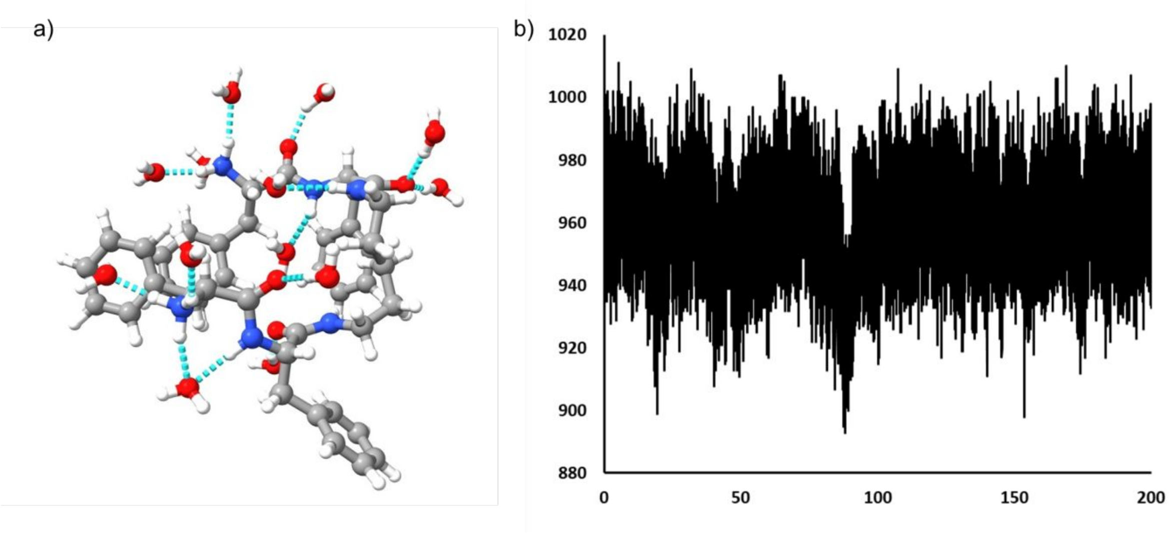

Figure S10. a) Hydrogen bonds formed between the compound and the surrounding water molecules, b) The total number of hydrogen bonds formed between all copies of the compound and water molecules throughout the simulation time. The x-axis represents time (ns), and the y-axis represents the number of hydrogen bonds.

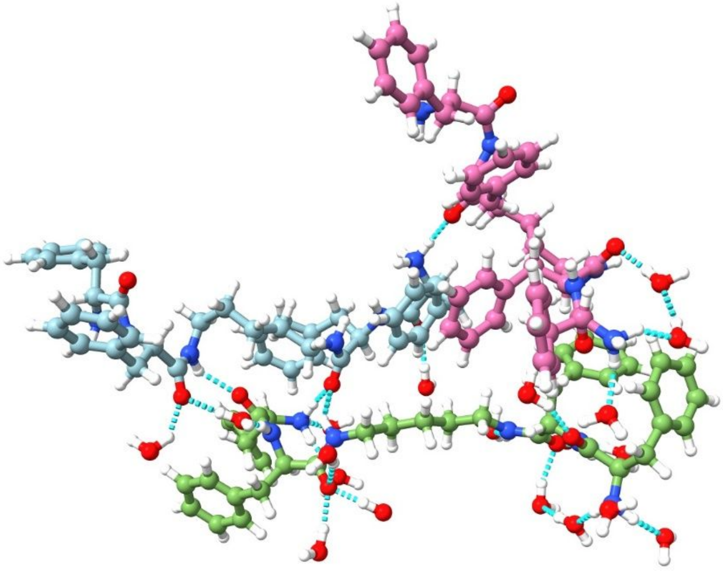

Figure S11. Bond energies calculation for intermolecular hydrogen bonding and with surrounding water molecules.

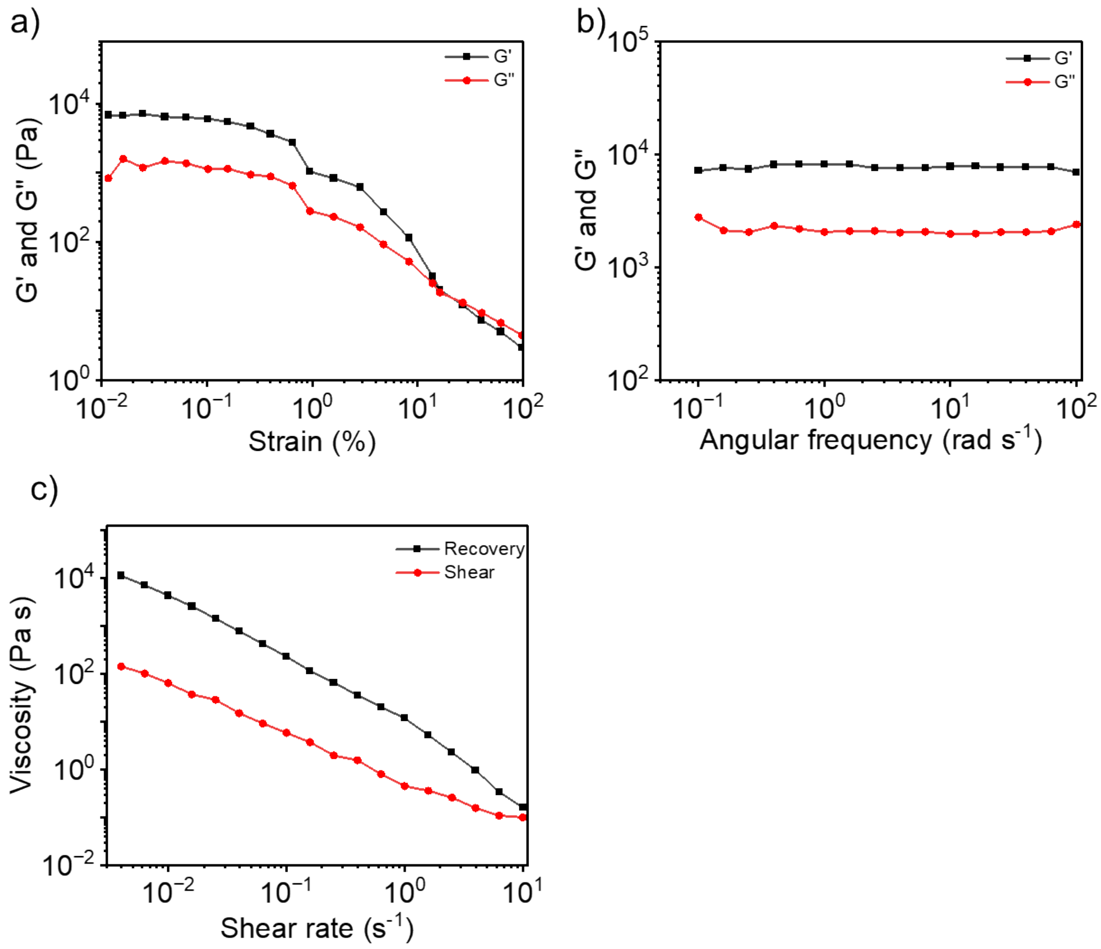

Figure S12. Rheological characterization of LFc_5FL hydrogel, a) Strain-dependent ($\omega = 10$ rad s^{-1}) oscillatory shear rheology of the peptide hydrogel (10 mg/mL), b) Angular frequency-dependent at a strain of 1 % oscillatory shear rheology of the peptide hydrogel (10 mg/mL), c) Shear-thinning and rapid recovery of the hydrogel by viscosity of peptide hydrogel at increased shear rate by continuous flow experiment.

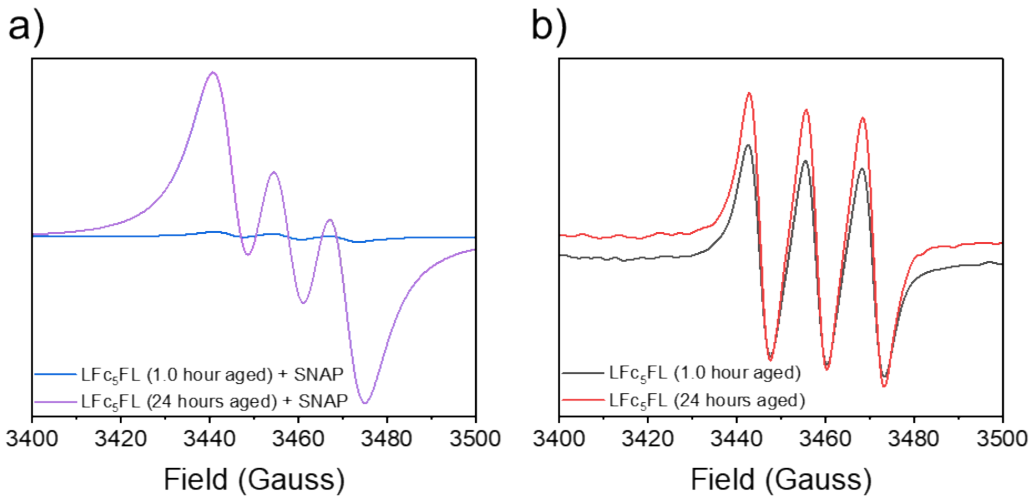

Figure S13. EPR Spectra of LFc$_5$FL (1mg/mL) hydrogel, a) with SNAP for NO release for NO generation, b) without SNAP for NO encapsulation from external source.

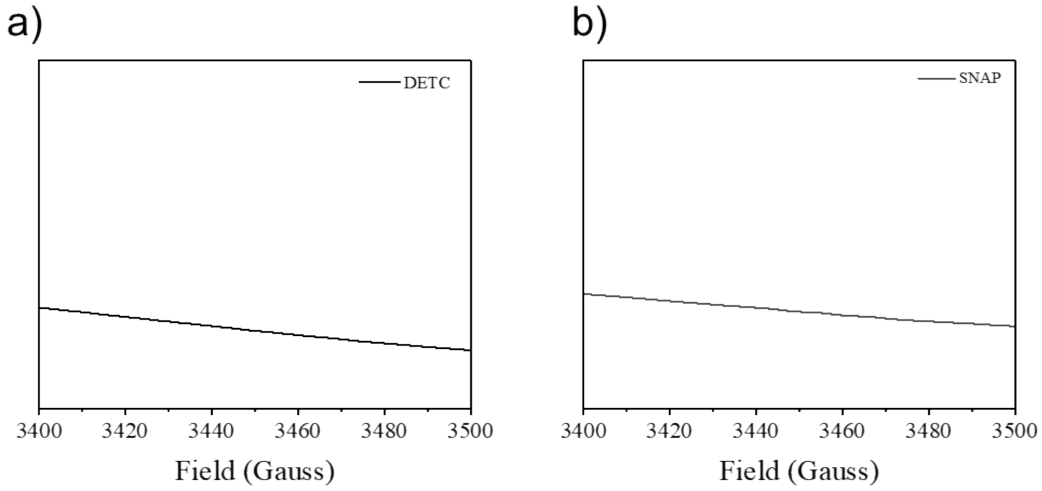

Figure S14. EPR Spectra of DETC (a) and SNAP (b)

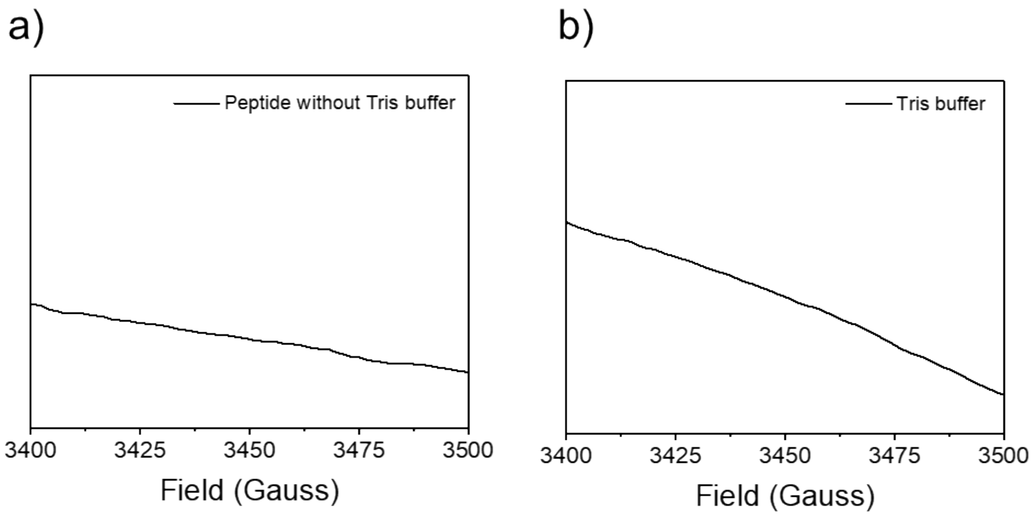

Figure S15. EPR Spectra of FFc_5FF without tris buffer (a) and tris buffer (b)

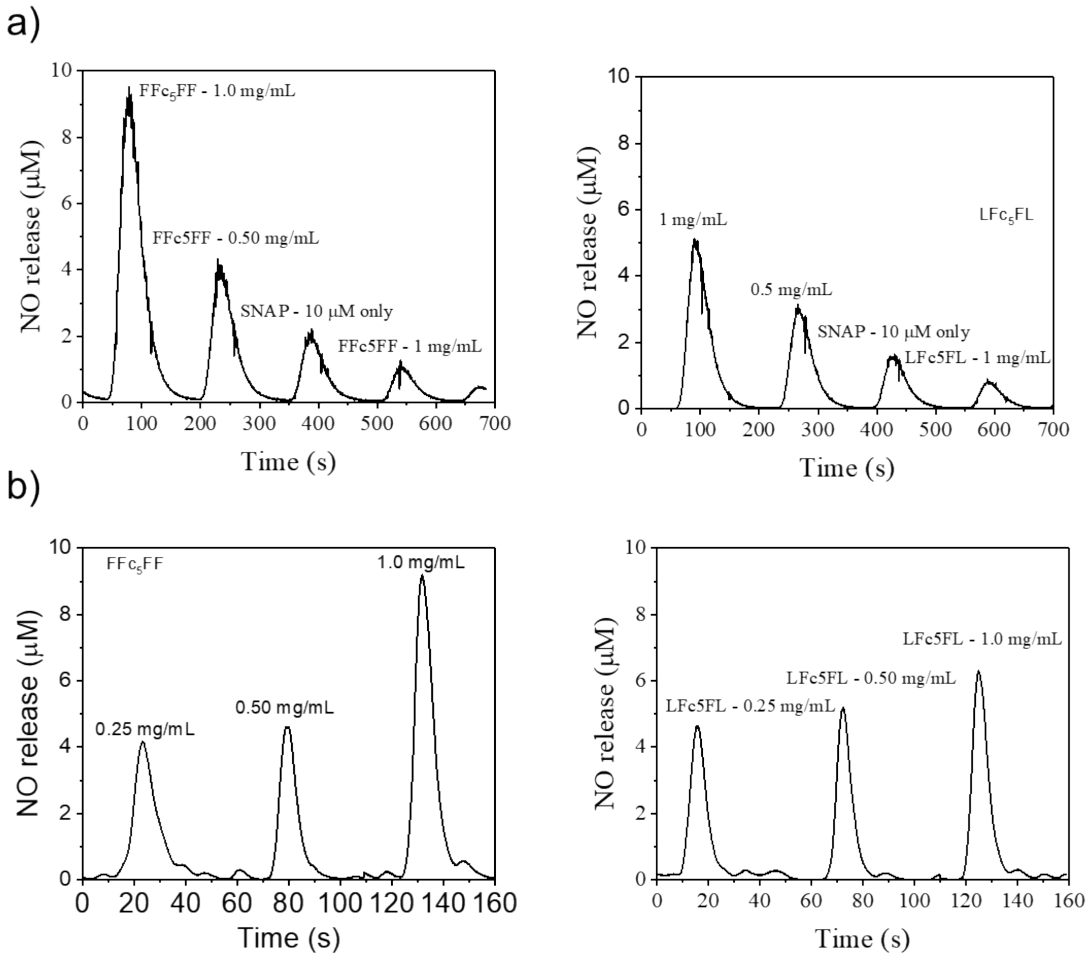

Figure S13. Chemiluminescence results for NO generation and encapsulation, a) the concentrations 0.5 and 1 mg/mL of FFc_5FF hydrogel (left) and LFc_5FL (right) nanofibrous scaffold show the catalytic activity with SNAP to generate NO, b) encapsulation of NO when stored from an external source in 0.25 mg/mL, 0.5 mg/mL and 1 mg/mL of FFc_5FF (left panel) and same concentration of LFc_5FL (right panel).

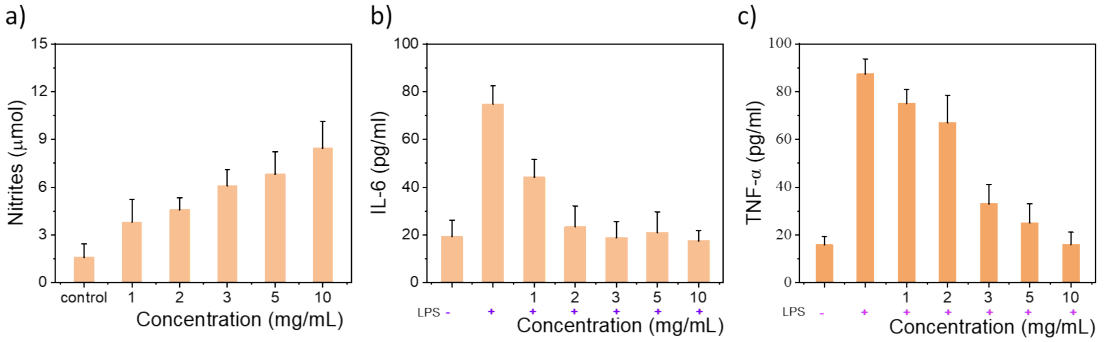

Figure S14. Anti-inflammatory studies of LFc$_5$FL, a) proinflammatory cytokines IL-6, and b) TNF-α release was measured using enzyme-linked immunosorbent assay (ELISA), c) the Griess assay measured the amounts of nitrites released from samples in LPS-induced RAW264.7. Data is expressed as mean ± SD (n-3).

9. References

[1] M. Parrinello, A. Rahman, *Journal of Applied physics* **1981**, *52*, 7182-7190.
